# Structure-Based Sequence Alignment of the Transmembrane Domains of All Human GPCRs: Phylogenetic, Structural and Functional Implications

**DOI:** 10.1371/journal.pcbi.1004805

**Published:** 2016-03-30

**Authors:** Vaclav Cvicek, William A. Goddard, Ravinder Abrol

**Affiliations:** 1 Materials and Process Simulation Center, California Institute of Technology, Pasadena, California, United States of America; 2 Minnesota Supercomputing Institute, University of Minnesota, Minneapolis, Minnesota, United States of America; 3 Departments of Biomedical Sciences and Medicine, Cedars-Sinai Medical Center, Los Angeles, California, United States of America; Tel Aviv University, ISRAEL

## Abstract

The understanding of G-protein coupled receptors (GPCRs) is undergoing a revolution due to increased information about their signaling and the experimental determination of structures for more than 25 receptors. The availability of at least one receptor structure for each of the GPCR classes, well separated in sequence space, enables an integrated superfamily-wide analysis to identify signatures involving the role of conserved residues, conserved contacts, and downstream signaling in the context of receptor structures. In this study, we align the transmembrane (TM) domains of all experimental GPCR structures to maximize the conserved inter-helical contacts. The resulting superfamily-wide GpcR Sequence-Structure (GRoSS) alignment of the TM domains for all human GPCR sequences is sufficient to generate a phylogenetic tree that correctly distinguishes all different GPCR classes, suggesting that the class-level differences in the GPCR superfamily are encoded at least partly in the TM domains. The inter-helical contacts conserved across all GPCR classes describe the evolutionarily conserved GPCR structural fold. The corresponding structural alignment of the inactive and active conformations, available for a few GPCRs, identifies activation hot-spot residues in the TM domains that get rewired upon activation. Many GPCR mutations, known to alter receptor signaling and cause disease, are located at these conserved contact and activation hot-spot residue positions. The GRoSS alignment places the chemosensory receptor subfamilies for bitter taste (TAS2R) and pheromones (Vomeronasal, VN1R) in the rhodopsin family, known to contain the chemosensory olfactory receptor subfamily. The GRoSS alignment also enables the quantification of the structural variability in the TM regions of experimental structures, useful for homology modeling and structure prediction of receptors. Furthermore, this alignment identifies structurally and functionally important residues in all human GPCRs. These residues can be used to make testable hypotheses about the structural basis of receptor function and about the molecular basis of disease-associated single nucleotide polymorphisms.

## Author Summary

G-protein coupled receptors (GPCRs) are a large superfamily of integral membrane proteins that share a characteristic 7 transmembrane helix fold. They detect various molecules outside of the cell and signal their presence to the inside of the cell. At least half of the 800 human GPCRs are potential drug targets, so understanding their structure and function is critical. Experimental structures are now available for at least one receptor from each GPCR class. The structure of the 7 helix fold is highly conserved even for receptors with very low sequence similarity. We analyze the available experimental structures and compare the common inter-helical contacts. Our analysis leads to a unified sequence-structure alignment of the GPCR superfamily that can then be used as the starting point for structure prediction of all other GPCRs. A key result of our analysis is a list of conserved contact residues and activation “hot-spots” residues that are critical for GPCR folding and function. We propose that mutations and natural variants of amino acids at these locations in the GPCRs can dramatically influence their activation state and alter intracellular signaling. This provides hypotheses for the molecular mechanisms underlying disease causing mutants for any GPCR.

## Introduction

### Structural revolution in the GPCR superfamily

G protein-coupled receptors (GPCRs) comprise the largest superfamily of integral membrane proteins, covering ∼3% of the human proteome. They mediate transmembrane (TM) signal transduction by allosterically facilitating information transfer across the cellular membrane in response to extracellular signals [1, 2]. The GPCRs are pleiotropic proteins responsible for sensing a diverse set of extracellular signals ranging from photons and small molecules (neurotransmitters, metabolites, odorants, tastants) to large oligopeptides (chemokines, incretins), and converting them into one or more intracellular signaling cascades. This critical role of GPCRs in cellular signaling makes them therapeutic targets in a large number of diseases, either due to their direct role in the pathophysiology of a specific disease or due to their ability to modulate a set of signaling cascades implicated in a disease [3]. Currently, about 30–50% of all drugs and 20% of recently FDA approved drugs act through modulating GPCR functions [4].

Experimental structures are now available for more than 25 different GPCRs covering all four major GPCR phylogenetic classes. Several GPCRs have been crystallized in complex with ligands, and some have been crystallized in active conformations, capable of coupling to G proteins or arrestins. Furthermore, efforts to crystalize most human GPCR proteins are underway [5]. The human *β*_2_ adrenergic receptor was crystallized in an active conformation in complex with the full heterotrimeric Gs protein, providing a snapshot of the conformational changes in both the receptor and the cognate G protein during GPCR activation [6, 7]. Rhodopsin has been recently crystallized with arrestin [8] providing the first detailed snapshot of the receptor before and during internalization. These new structures inspire a full spectrum of mechanistic studies into the GPCR biology, and will guide the functional understanding and pharmacological targeting of these receptors [9–11].

**Fig 1:**
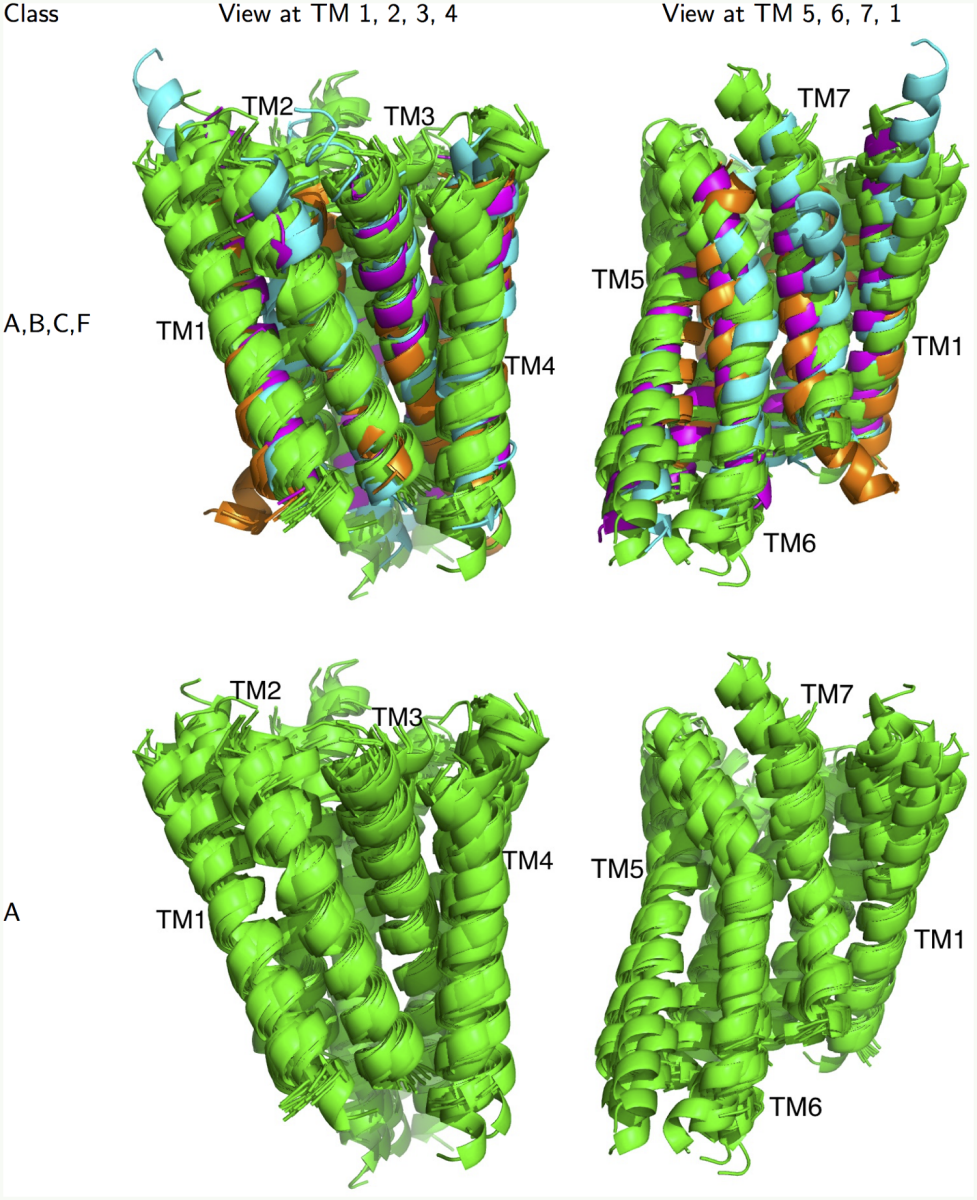
TM domains of the available crystal structures. Top: Two views of the 24 inactive crystal structures from classes A, B, C, and F (aligned to *β*_2_) show the general GPCR fold of the transmembrane (TM) bundle. Class A in green, class B in blue (CRF1, GLR), class C in orange (MGLU1, MGLU5), class F in magenta (SMO). Bottom: Same views for only the 19 inactive class A structures showing the highly conserved class A TM fold. A detailed view of the conserved hydrogen bonding networks is shown in S1 Fig.

The cellular membrane partitions a GPCR protein into 3 domains: extracellular, transmembrane, and intracellular [12, 13]. The N-terminus and three extracellular loops (EC1, EC2, EC3) lie outside of the cell; seven transmembrane (TM) helices (TM1-7) span the membrane; and inside the cell, there are three intracellular loops (IC1, IC2, IC3) together with the C-terminus, which typically contains a shorter helix 8 resting parallel to the membrane. The N-terminus, intracellular loops, extracellular loops, and C-terminus can have very different lengths, not just across the major GPCR classes but also within a GPCR class [14]. The loop regions are flexible and display different conformations among the known GPCR crystal structures. However, the packing of the TM helices is remarkably well conserved (Fig 1) even for proteins with very small sequence similarity (down to 20%, S2 Fig).

For class A GPCRs this is not a surprise, because several amino acids forming inter-helical hydrogen bonds are highly conserved. Indeed, the conserved residues directly correspond to each other in the structural alignment, as can be seen on S1 Fig. Within class A, the sequence similarity between the corresponding transmembrane regions for the receptors with experimentally determined structures is 35–100% (S2 Fig), which is high enough that a sequence alignment algorithm (such as Clustal Omega) typically aligns the transmembrane regions correctly. However, the sequence similarity between class A and other classes is so low, typically 20–30%, that sequence alignment algorithms generally fail.

At the time of this study, there were 5 non-class A GPCR structures available. Fig 1 shows that when these structures are aligned to class A using the GRoSS alignment (introduced below in the methods section), their transmembrane helices are *in the same fold as that of class A GPCRs*. A certain degree of similarity was expected for the intracellular face of the receptor, where they couple to the same set of G proteins and arrestins, but the similarity seems to be present in the full TM bundle. With this comparison of the structures of GPCRs from different classes, we are now able to examine the features that survived the evolutionary divergence in sequence potentially on the basis of their functional importance, enabling us to gain insights into the general features of the GPCR structural fold.

### Transmembrane domains as a basis for modeling conformational diversity

Computational protein folding works best for small water soluble proteins and it has proved effective for up to about 100 residues [15]. The energy models used for soluble proteins have been extended to include approximations of the membrane environment [16, 17]. However, the accuracy of these methods is not sufficient for drug design, partly due to the size of many membrane proteins. GPCRs are large proteins with more than 300 residues, making them difficult to model with standard protein modeling methods. Recent assessments [18–20] of the GPCR structure prediction community showed that approaches based on homology models remain the most successful [21–23].

Homology modeling requires accurate sequence alignment to existing structures, which fails completely for alignment between classes. Within each class, sequence alignments typically conserve key motifs, but they often lead to gaps in the transmembrane helices, which indicate serious problems. Recently, Isberg et al. [24] studied in detail the available crystal structures and obtained pairwise sequence alignments from the highly conserved 7 helix fold by aligning individual TM domains of two structures at a time. Their alignment leads to many single amino acid gaps, which correct for non-matching bends and turns of the TM helices that are often caused by mismatched Pro or Gly. Nevertheless, it is not possible to predict these gap positions with sequence information alone, without the knowledge of the correct structure. Therefore it is not possible to extend their alignment to other GPCRs. Our goal was to construct a sequence alignment of the TM domains of **all human GPCRs**, that would be suitable for 3D structure prediction. Thus we developed a method that avoids the gaps in the TM regions.

We can consider the common GPCR structural fold to be formed by alpha-helices, allowing us to think about the structural relationships in terms of rigid helices. Past studies [25, 26] used the rigid transmembrane helices to compare the different GPCR receptors. The comparative analysis of available GPCR structures informs structure prediction methods (such as in [27]), where 3D rotations and tilts of the rigid helices are used to sample conformations of the protein in the membrane. Moving the whole helix at once can overcome large potential barriers and sample possible inactive and active states. Better definitions of the GPCR fold and its activation mechanism can help to reduce the number of coordinates needed for modeling.

### Integrating sequence, structure, function

Several studies have analyzed inter-helical contacts [28], ionizable networks [29], or developed a phylogenetic analysis of class A GPCRs [30], but no one has yet reported a GPCR superfamily-wide sequence alignment that avoids gaps within the TM domains. The available GPCR structures in the PDB cover a wide sequence space, with some structures showing different ligands bound to the same receptor or the same ligand bound to different receptors, and some structures showing different conformations for the same receptor. The fact that the seven helix fold is conserved across divergent sequences can teach us about the nature of the GPCR fold and its features.

In this work, we first generate a **structure-based alignment** of the TM regions of the available 25 structures by maximizing the number of the corresponding inter-helical contacts. We use chemically nonspecific contacts instead of conserved residues, since, even though the amino acids are not conserved across this diverse superfamily, many **inter-helical contacts are conserved** across all GPCR classes as will be shown below. Our structural alignment is extended to include all human GPCRs by sequence alignments of small subgroups of similar receptors. The final GPCR sequence-structure alignment (GRoSS) includes the TM regions of **all** 817 human GPCRs. The GRoSS alignment is then used to generate a phylogenetic tree for all human GPCRs, which correctly distinguishes the different receptor classes, suggesting that the functional information about different GPCR classes, that are usually characterized by variable length N/C-termini and loop regions, is in fact encoded at least in part within the GPCR TM domains.

This observation that the TM domains and their relationships capture the similarities between the rather disparate set of GPCRs, has broad implications in terms of simplifying and enhancing the connection between receptor sequence and function. We identified 23 Conserved inter-HelIcal COntacts (CHICOs) that define the general GPCR structural fold. Furthermore, our structural comparisons of inactive and active conformations lead to the identification of 15 Native ACtivation “Hot-spOt” residues (NACHOs). We expect that mutations of the structurally important CHICO and NACHO residues will dramatically affect receptor function, and may be responsible for many diseases. Cross-checking these residues with available GPCR mutation databases (Uniprot [31], NAVA [32], TinyGRAP [33]), which contain disease/function association, has allowed us to identify several deleterious receptor mutations that are found at or adjacent to the “hot-spot” residues as shown later in the results section. Using the GRoSS alignment, we have mapped these important positions to all GPCRs leading us to suggest new testable hypotheses about molecular mechanisms behind natural or man-made mutations. We expect that this will create new rational drug discovery opportunities for efficacious therapeutics that minimize side-effects.

## Methods

In this section we describe our procedure for sequence alignment of the TM domains of all 817 human GPCR proteins. The resulting alignment is available in S2 Table, and in fasta format in S1 File.

### Human GPCR classification

After the Human Genome Project was completed, Fredriksson et al. [34] performed detailed phylogenetic analysis of GPCRs developing the GRAFS classification system [34, 35], which identified 5 main groups of receptors: Glutamate, Rhodopsin, Adhesion, Frizzled/Taste2, and Secretin. The Rhodopsin family is the largest and was partitioned into four subgroups: *α*, *β*, *γ*, and *δ*. Olfactory receptors were attached to the *δ* branch. The bitter taste receptors (Taste receptors type 2, Taste2, or TAS2Rs) were grouped with the Frizzled family.

Currently, the list of receptors is maintained by the International Union of Basic and Clinical Pharmacology (IUPHAR) [36], which also keeps track of known endogenous ligands and signaling mechanisms. IUPHAR identifies 6 main classes A-F: Four of them, class A (rhodopsin-like receptors), class B (secretin and adhesion family), class C (glutamate receptors), and class F (frizzled receptors) contain human receptors. Classes D, E are part of the classification that includes other species, but contain no human orthologs. In contrast to GRAFS, secretin and adhesion groups have been merged since their sequences align well. The bitter taste receptors (Taste2, or TAS2Rs) and Vomeronasal receptors (VN1Rs) are beginning to be considered similar to class A, in contrast to the GRAFS classification, which is what we also find in the phylogenetic analysis presented below.

### GPCR database

We extended the list of human GPCRs of Fredriksson et al. [34], with the proteins considered by the GPCR Network [5] and by IUPHAR [36]. Furthermore we added all sequences annotated as human GPCR proteins in the Uniprot database [31]. Altogether we collected 836 candidate sequences.

Most of these proteins have been assigned to a class by previous studies [34, 36]. For sequences with unknown class, we first searched for related proteins by running BLAST, and then we aligned them against the candidate class with the program Clustal Omega [37]. If the sequence aligned without large gaps in the TM regions for any of the classes, we assigned the sequence to this class. The following 11 proteins might be GPCR proteins, but they do not align well to any of the classes, thus we ignore them in the further analysis (listed as Uniprot accession numbers, ACs):

P51810, Q5T9L3, Q5VW38, O60478, Q86V85, Q86W33, Q8N3F9, Q8NBN3, Q96K49, Q96N19, Q9NPR9.

The following 8 sequences are most likely pseudogenes, because they are missing one or more TM domains:

A6NFC9, Q32VQ0, Q8NGA4, Q8NGU1, Q8NGY7, Q8TDU5, Q96P88, Q99463.

We kept Q9P1P4 (TAAR3) which is a pseudogene in humans but functional in rodents, and we kept Q49SQ1 (GPR33), which may be functional in some people. A curious case is the protein GPR157 (Uniprot AC Q5UAW9), which is most similar to class B, but its TM1 has a gap in the alignment to class B. However, the TM1 aligns well to the class A TM1, so this protein appears to be a hybrid between these two classes.

Table 1 summarizes the count of all the sequences that we kept and S2 Table lists their Uniprot ACs. In total there are 817 candidate human GPCRs, 399 of which are non-olfactory. If the sequences in class A were present in the analysis of Fredriksson et al. [34], then we kept the subgroup labels *α*, *β*, *γ*, *δ*, otherwise we labeled them as *A-other*.

**Table 1:** Number of GPCR sequences by class. The total number of candidate human GPCR sequences that were considered are listed. The full list of Uniprot ACs is in S2 Table.

88	A*α*	16	B	5	Vomeronasal
33	A*β*	22	C	25	Taste2
57	A*γ*	11	F	11	Other
58	A*δ*	33	Adhesion	8	Pseudogene
51	A-other	418	Olfactory		

### Available crystal structures

At the time of this study, crystal structures of 25 GPCRs were available (19 of these for human sequences): 4 in both active and inactive conformation [6, 38–44], 1 in active only [45], and 20 in inactive conformation only [46–66]. When multiple crystal structures of the same protein were available, we used the one with the best resolution or the one with the best-defined transmembrane alpha helices. The PDB IDs of the structures used in analysis are listed in S1 Table.

Well-defined TM helices are a prerequisite for our analysis, however, different criteria have been used for annotating helices in different PDB files. Many of the transmembrane helices contain bends, and sometimes the helix termination is not well defined. We define the extent of each transmembrane helix as the residues positioned in the membrane (as placed by the Orientations of Proteins in Membranes (OPM) database [67]) extended until the end of the alpha helix by the DSSP secondary structure determination [68]. The helices were manually inspected and only a few manual corrections were needed. The final TM lengths used are displayed in S2 Table.

### Alignment by minimizing RMSD is not unique

As suggested by Fig 1, the transmembrane helical bundles of all the GPCR structures display the same structural fold (same relative position of all seven helices). The main basis for some published structural comparisons of classes A and B, C, F was an iterative structural alignment implemented by the program ICM-Pro [69] used for GLR, MGLU1, and SMO. Also, MGLU5 was aligned to class A with an iterative algorithm (SSM algorithm [70]) and CRF1 was aligned to class A manually. The iterative structural alignment algorithm, removes from the alignment all atoms that are too far in the previous rounds. This works relatively well, but the exact sequence pairing is not uniquely defined and depends on cutoff parameters. In many cases it leaves an ambiguity of ±4 residues (1 helical turn).

In order to remove this ambiguity and to determine which alignment would minimize the root mean squared deviation (RMSD) of the full TM bundle, we start from an approximate structural alignment, and try all nearby sequence alignments (±1 helical turn on each helix). For each sequence alignment, we first select the maximal overlapping lengths of the 7 TMs. Then we compute the C_*α*_ RMSD by evaluating the least-squares superposition of the corresponding C_*α*_ atoms. But minimizing only RMSD does not necessarily lead to an optimal alignment. For several cases in class A, we find TM alignments that have a lower RMSD than the alignment that conserves the correspondence of the same Ballesteros-Weinstein (BW) residue positions [12] (some BW residue positions in each TM domain are expected to be conserved at least within a GPCR class and hence align well). The reason for this is that the extracellular ends of the helices sometimes have significantly different tilts, making the most tilted helix dominate the RMSD measure. To avoid these issues with the RMSD measure, we instead look for an alignment, which maximizes the number of conserved inter-helical contacts. A contact is defined to be conserved if the residue pair in the contact has the same BW numbering in the two structures being compared. For example, if structure X has an inter-helical contact between residues 2.45–3.42 and structure Y also has an inter-helical contact between residues 2.45–3.42, then this contact is defined as conserved in the two structures. The BW numbering of residues in structure X and structure Y implicitly assumes that the sequence for structure X and the sequence for structure Y are aligned to superimpose residues with the same BW numbering. This measure accommodates the scenario commonly seen in GPCRs and described below, where residues may not be conserved but the corresponding structural contacts have been conserved during evolution.

### Class A conserved contacts

**Fig 2:**
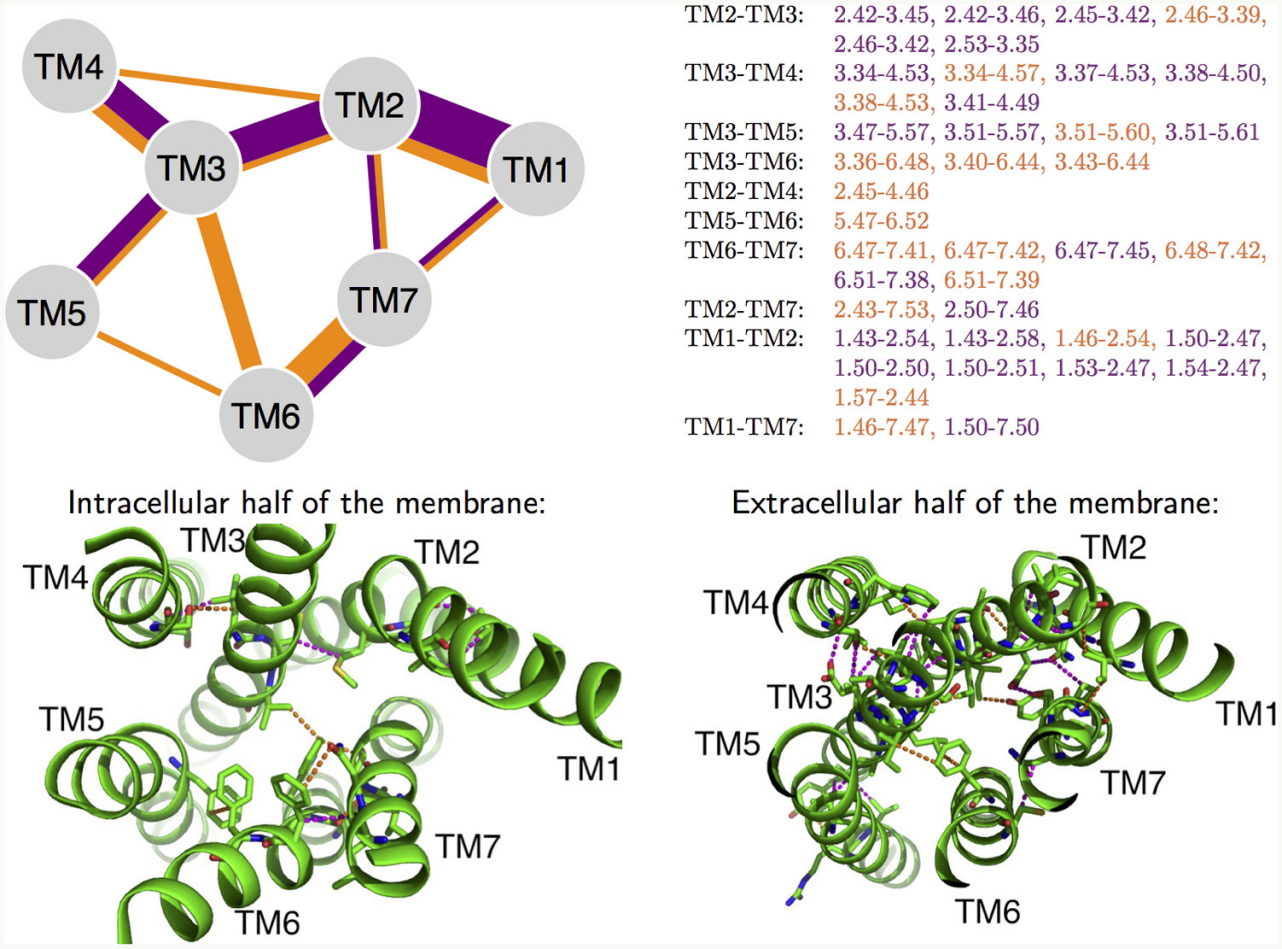
Conserved inter-helical contacts. Top left: Diagram of 40 conserved inter-helical contacts (CHICOs) present in at least 23 out of 24 studied class A structures. The contacts common to all classes are shown in purple, and contacts present only in class A in orange. Top right: List of these contacts in Ballesteros-Weinstein numbering scheme. Bottom: Extracellular view of the same contacts in the *β*_2_ crystal structure. The contacts in the inner and outer half of the membrane are shows on the left and right respectively.

In class A, the analysis of inter-helical interactions typically focuses on hydrogen bonds, since many of the hydrogen bonding residues are highly conserved. In order to compare receptors from different classes, which have poor sequence conservation, we compare inter-helical contacts ignoring their chemical nature. We use the definition of an inter-helical contact as in [28]: any two heavy atoms from different TMs that are closer than the sum of their van der Waals radii plus 0.6 Å. The inter-helical contacts, which are present in almost all class A structures, are shown in Fig 2. This list is very similar to the contacts found by [28], but there are minor differences caused by using a different set of crystal structures.

We briefly compare this list of chemically unspecific contacts to the conserved hydrogen bonds. Within class A, the focus is often on two conserved networks of hydrogen bonds (shown in S1 Fig):

**4-2-3**: W4.50 ↔ S/N/T2.45 ↔ S/N/T3.42

**1-2-7**: N1.50 ↔ D2.50 ↔ N7.49.

Fig 2 shows that the network 4-2-3 is well conserved across classes. In particular, the contact 2.45–3.42 is present in all classes, and there are many conserved contacts in its immediate vicinity, such as 2.42–3.45, 2.42–3.46, and 2.46–3.42. The contact between TM 3 and 4 is also well conserved, as the highly conserved bulky residue W4.50 leans on A3.38.

The network 1-2-7 also has many conserved contacts, including N1.50-D2.50, but interactions with N7.49 are not conserved. Even in class A, N7.49 interacts with the other residues of this hydrogen bonding network indirectly through a water molecule. Still, the side chain packing in the regions where the helices are close together is important for structural stability. Conserved water-mediated interactions could be included in future analysis, after enough crystal structures resolve them. For class A it is clear that using BW numbering greatly facilitates sequence and structure comparisons. Next we will see how these ideas can be extended to other classes.

### Sequence alignment across classes based on common contacts

To compare other GPCR classes with class A, we first find the inter-helical contacts of all the current crystal structures. Each structure has about 200 contacts. For any pair of structures and a given sequence alignment, we compute the number of conserved inter-helical contacts. We consider all possible TM sequence alignments that have no gaps (±10 residues from the starting sequence alignment). For class A, the number of common contacts between any two structures is maximized by the alignment that preserves the BW numbering.

To translate the BW numbering into classes B, C, and F, we start with a sequence alignment corresponding to an approximate initial structural alignment to class A. For each non-class-A structure, we then try all possible adjustments to BW *n*.50 residues (±10 residues, again with no gaps) on each helix and count the number of common contacts with each of the 24 class A structures. Table 2 shows the alignments with the highest cumulative number of the common inter-helical contacts.

**Table 2:** Selection of the alignment between class A and classes B, C, and F. This table shows the selection process for assigning BW.50 residues to non class A proteins. Shifting BW.50 residue on each helix renumbers the relative BW numbers, effectively changing the labels of contacts observed in these proteins. Subsequently, the number of common contacts each structure shares with the class A structures changes for different BW residue assignments. The second rightmost column shows the cumulative number of contact occurrences among the 24 class A structures (including active conformations). The BW assignment with the highest number of contacts is selected (except for MGLU5, see text). The selected alignment is in bold.

Class	Protein	BW#							Common contacts	RMSD Å
		1.50	2.50	3.50	4.50	5.50	6.50	7.50		
B	CRF1	**L134**	**F162**	**L213**	**W236**	**V279**	**L329**	**S360**	**2212**	**3.11**
		L134	F162	L213	W236	V279	I325	A363	2084	3.61
		L134	F162	L213	W236	V279	I325	S360	2081	3.35
	GLR	**L156**	**F184**	**L249**	**W272**	**A314**	**V364**	**A397**	**1972**	**2.93**
		L156	F184	L249	G273	A314	V364	A397	1913	2.94
		L156	F184	L249	M276	A314	V364	A397	1876	3.11
C	MGLU1	**T607**	**I638**	**I682**	**I714**	**L763**	**A800**	**L827**	**2017**	**3.02**
		T607	I638	I682	S711	L763	A800	L827	2012	3.24
		T607	I638	I682	S711	C767	A800	L827	1873	3.41
	MGLU5	T594	I625	A669	F698	L750	A787	L814	1974	3.36
		**T594**	**I625**	**A669**	**I701**	**L750**	**A787**	**L814**	**1954**	**3.17**
		T594	I625	A669	F698	L750	I784	L814	1820	3.64
F	SMO	**T245**	**F274**	**W339**	**W365**	**V411**	**I465**	**S533**	**2358**	**3.00**
		T245	F274	W339	W365	V411	C469	G529	2311	2.98
		T245	F274	W339	W365	V411	C469	S533	2248	3.02
		…								
		T245	F274	W339	W365	V411	S468	I530	1827	3.04

In class A, the BW *n*.50 (*n* = 1 to 7 denotes the TM) residues correspond to the most conserved residues in each TM. After the projection of the BW numbering to the other classes, the *n*.50 residues are not necessarily the most conserved within each class. Moreover, they correspond neither to class B specific Wooten numbering [71], nor the class C specific Pin numbering [72], nor the class F specific numbering [65], which only define labels *n*.50 for residues that are the most conserved within the respective classes. In the context of Isberg et al. [24], our *n*.50 can be referred to as *n*.50*a* and uses class A residues as a reference to number TM residues for GPCRs from all classes. This unified numbering scheme enables a view of structurally conserved or structurally similar positioned residues across all GPCR classes because conserved residues in individual GPCR classes have no structural similarity or positioning in the GPCR structural fold in cross-class comparisons.

In terms of absolute sequence alignment of TM domains, our Class B (CRF1, GLR) alignment agrees with the alignment suggested in [62], which was obtained by an iterative structural alignment. Similarly, our alignment for class C (MGLU1, MGLU5), agrees with the suggested alignment in [63]. For MGLU5, we chose the second highest scoring alignment to make the alignment consistent with the MGLU1. This choice was checked visually and the corresponding residues are in a more similar position in our selected alignment.

Our alignment for the SMO receptors is the only one that differs from the published alignment to class A presented with the crystal structure [65]. This published alignment corresponds to the last row of Table 2, and it differs from our best alignment (fourth to last row of Table 2) in TM6 and TM7 by 3 residues each. The RMSD difference between this published alignment and our alignment is small, however, our alignment results in ∼500 more common structural contacts than the former alignment. Helix 7 of the SMO receptor does not have a proline residue, and so it is missing the kink that is typical for class A GPCRs. There are many inter-helical contacts in the extracellular part of the TM7, so that our chosen alignment gives a good spatial correspondence for the larger part of helix 7. We have presented a well-defined protocol for structural-sequence alignment. As more experimental structures become available, this protocol can refine the alignments where needed, which might occur especially for classes with a small number of known crystal structures.

This sequence alignment was used to generate the structural alignment shown in Fig 1. The sequence similarities between the TM domains of the crystal structures are shown in S2 Fig, and the TM domain RMSDs are shown in S3 Fig. Sequence similarities correlate reasonably well with the RMSDs (structural differences). Note that we define percent similarity as the fraction of similar residues, where two residues are similar if their BLOSUM62 [73] (or GPCRtm [74]) matrix entry is positive.

### GRoSS: Extension of alignment to all known GPCR sequences

We extend the structure-based alignment derived above to all human GPCRs by anchoring each subfamily to the correct crystal structure. As a guide for the quality of the sequence alignment, we check for the presence of any gaps in the transmembrane regions. The approximate positions of the TM regions are already annotated in the Uniprot database as predicted by the TMHMM program [75]. These predictions are quite noisy, and even for similar proteins that align well, they can differ by 5–8 residues and sometimes even misclassify a TM. However, for multiple sequences the overall trend clearly identifies the approximate TM location and allows us to judge the quality of the alignment of multiple sequences. If there are gaps in the TM regions, the alignment cannot be used to successfully create homology models.

First, we try to align directly all 817 sequences of the GPCR superfamily using a multiple sequence alignment program Clustal Omega [37]. However, the overall sequence conservation is very low, and the resulting alignment has many large gaps even within TM domains. Some highly conserved residues end up aligned incorrectly. In order to avoid this problem, we aligned class A sequences separately (705, including olfactory). Again the resulting alignment has large gaps even in the TM region. It seems that the large variability of the loop region is what confuses the alignment algorithm.

Fortunately, we find that sequences in individual subgroups can be aligned using Clustal Omega without large gaps in the TM regions. We take these individual subgroup alignments and fix them into a *profile*—a multiple sequence file for which aligned columns are kept fixed, and from which the hidden Markov model (HMM) is computed. We then align any two profile HMMs to see how similar are the two groups. A profile alignment of A*α* to each of the other class A groups (A*β*, A*γ*, A*δ*) has no gaps in the TM regions, and also gives the correct alignment of the BW.50 residues.

The multiple sequence alignment of the group A-other showed gaps in the TM regions for several proteins (Uniprot ACs: Q96P67, Q8TDU6, Q16570, Q86SM8, Q9NS66, Q9NS67, P60893, Q86SM5), so a separate profile was created for these sequences. After the split, both profiles of the A-other proteins aligned separately to the A*α* profile without any gaps in the TM region, which anchors them to the class A alignment. Similarly, the profile of olfactory receptors (both tetrapod and fish-like) aligned to the A*α* profile without any gaps in the TM region, which anchors the olfactory profile to the class A alignment. The Vomeronasal and Taste2 groups were more problematic, and are discussed in the following section.

The profile of the adhesion class aligns well to secretin class (original class B). Class B is aligned to class A using the structural analysis described above. Aligning profiles of A*α* and B does not yield meaningful alignment, because the TM regions are offset and there are many gaps in the TM regions. Similarly, aligning classes A and C or A and F does not yield meaningful alignment, and again structural alignment was used for these cases.

Once the alignment is fixed, the TM lengths for new proteins can be predicted to be the average TM lengths from the available structures in the same class. For example, for sweet taste receptors (TAS1) the predicted TM length is the average TM length of GMR1 and GMR5; and for bitter taste receptors the average TM lengths of the 20 class A structures. These are meant to be the best initial guesses.

The complete listing of all the 817 human GPCR proteins is shown in S2 Table. The alignment of each helix is determined by the provided BW.50 residue. Expected TM range is also provided and it is estimated as the average TM region of the known crystal structures from the same class. For easy viewing, we provide the same alignment also in the fasta format together with the annotations of TM range and BW residues in Jalview [76] format in S1 File. The GRoSS alignment was also compared to alignments obtained by two other methods: HMM-HMM [77] and GPCRDB [24, 78]. This comparison is described in S1 Text.

### Bitter taste and vomeronasal receptors

Bitter taste (Taste2, TAS2R) and vomeronasal receptors are small groups of receptors that do not easily align to the profiles for classes A-F, and so their classification has not been unique. While IUPHAR assigns the bitter taste receptors into the class A, Singh et al. [79] points out the lack of conserved amino acids between the two.

**Fig 3:**
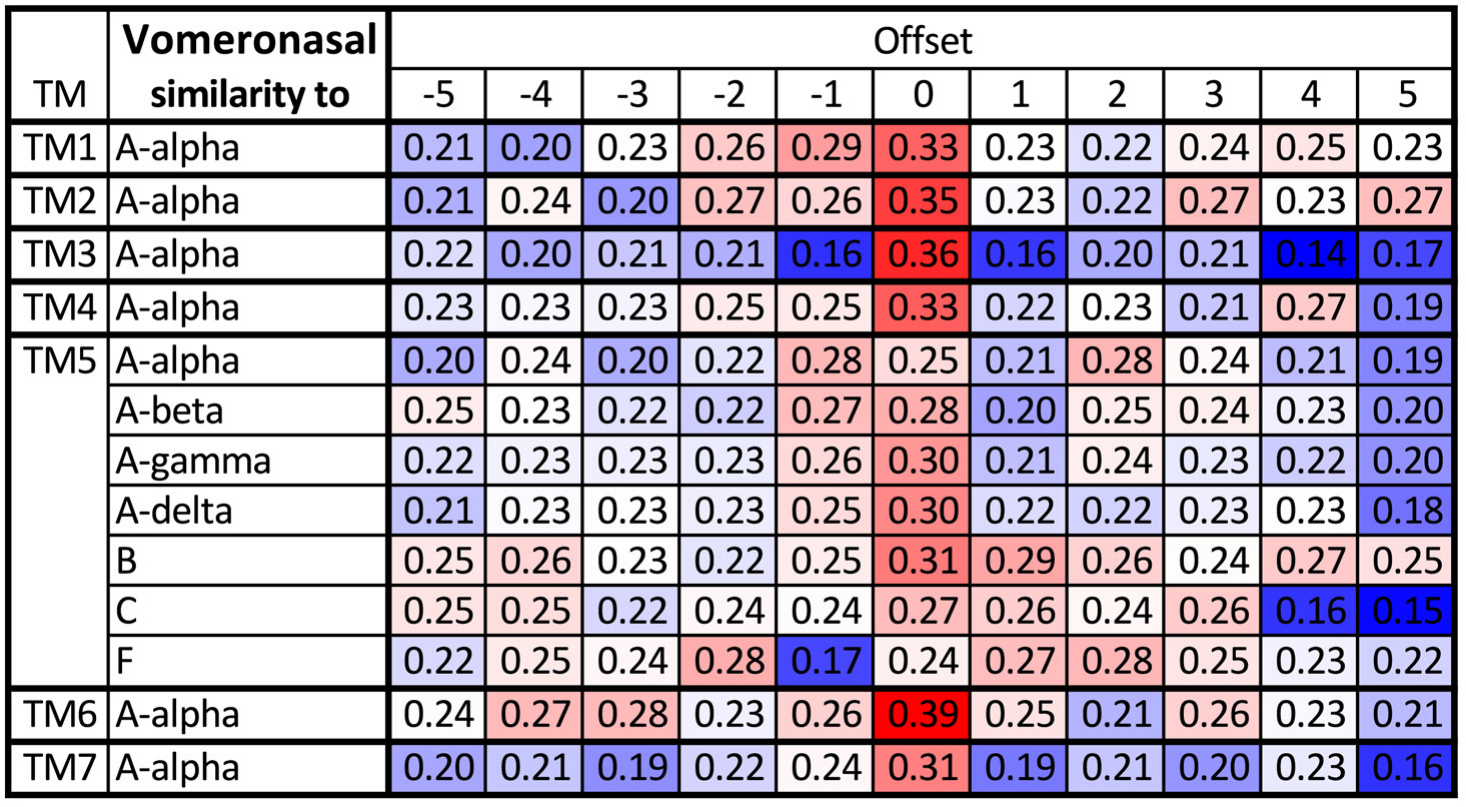
Testing the robustness of the alignment of the Vomeronasal receptors with the other groups. The table shows similarity between TMs averaged over all pairs of sequences formed from the two groups (red denotes high similarity, blue low similarity). For most TMs the optimal choices agree with the optimal alignment to A*α* (full table in S5 Fig); all combinations are shown only for TM5. The same table but using the GPCRtm substitution matrix [74] instead of BLOSUM62 is shown in S7 Fig. GPCRtm was developed in particular for GPCR proteins, but in this case both matrices result in the same alignment.

The profile of the vomeronasal group aligns better with class A*α* compared to classes B and C, but there is still a gap of length 2 near the center of TM5. We remove the gap in such a way that the residue, which aligns with 5.50 stays fixed. To check that this is indeed the best alignment we explore small changes in the alignment by shifting individual TM by up to ±5 residues. In Fig 3 we see that for TMs 1 to 4, our current alignment gives the highest sequence similarity with A*α*, so the alignment of these TMs is correct. However, for TM5, the alignment shifted by -1 or +2 residues gives higher similarity with A*α*. Nevertheless, the similarity with groups A*β*, A*γ*, A*δ*, and B is the highest for our current alignment. We therefore keep the current choice.

**Fig 4:**
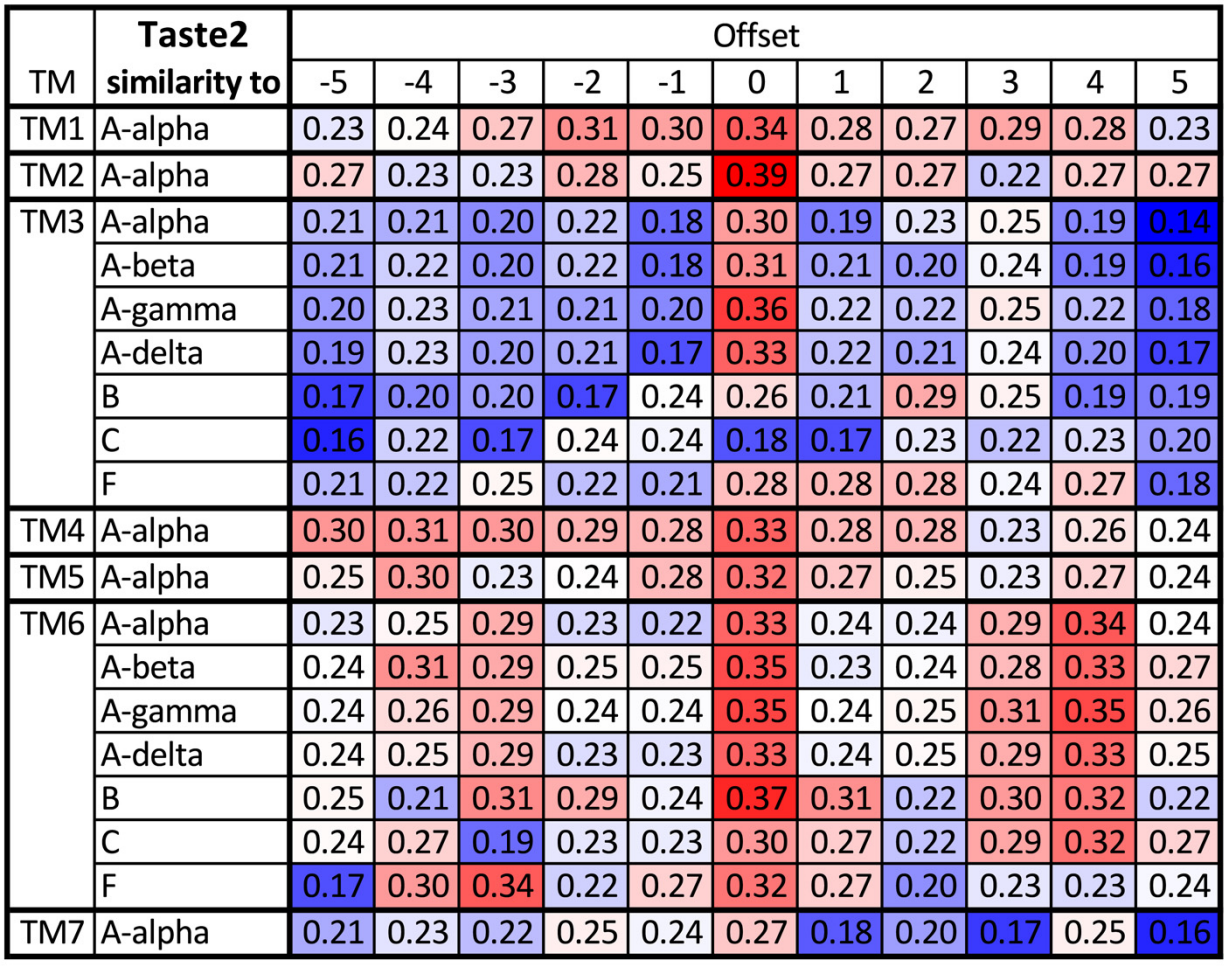
Testing the robustness of the alignment of the Taste2 receptors with the other groups. The table shows similarity between TMs averaged over all pairs of sequences formed from the two groups (red denotes high similarity, blue low similarity). For most TMs the optimal choices agree with the optimal alignment to A*α* (full table in S6 Fig) only TM6 shows a second possible alignment at offset +4. The same table but using the GPCRtm substitution matrix instead of BLOSUM62 is in S8 Fig. Again, both matrices result in the same alignment.

We performed a similar analysis for the Taste2 receptors, for which adjustments were necessary. The profile alignment of Taste2 with A*α* has some gaps, but it is still the best alignment (i.e., it has the fewest gaps) compared to aligning to classes other than class A. TM3 has two gaps in the alignment: a gap of length 4 in the middle of TM3, and a gap of length 5 at the DRY motive. As the first iteration we kept the alignment fixed on residue 3.50, then we computed the similarity to other groups for ±5 residue shifts. The shift by +3 residues gives better similarity and so it was kept. See Fig 4 for the computed similarities after the shift has been made. All class A subclasses favor this new choice, as the highest similarity has offset 0. Class B would favor shift by 2 residues, but the similarity is less than 30%.

TM4 has low sequence similarity, and in particular the highly conserved Trp is not present in Taste2. Again as a starting point we kept the alignment at 4.50, but later had to adjust it by 4 residues. Fig 4 shows the similarity after this shift has been made. For TM4 the similarity is only slightly higher at the new best offset than at nearby offsets.

Taste2 TM6 showed the partially conserved motif IYFLS, with S being aligned to P6.50, which we kept as an initial try. This choice is kept in Fig 4. However, we see that an offset of +4 residues, which corresponds to a one turn shift (the motif IYFLS aligns Ile with P6.50), also gives high similarity. Based solely on sequence similarity we cannot distinguish which alignment is better, and therefore both cases should be considered when building homology models and energy of the resulting structures should be used as a guide to select the best choice. These alignments will be revisited when the first experimental structure of one of the Taste2 receptors is determined.

### Computation of the phylogenetic tree

We compute the similarity for each pair of sequences using the weights from the BLOSUM62 matrix (two residues are considered similar if their BLOSUM62 matrix entry is positive), and use the similarities as a distance metric to cluster the proteins. We used the unweighted pair-group clustering algorithm (implemented in Jalview [76]), which iteratively extends clusters by finding a non-member sequence with the lowest average dissimilarity over the cluster members. The phylogenetic tree constructed by this clustering algorithm was visualized using the Iterative Tree of Live toolkit [80].

### List of natural variants

S3 Table lists all 2449 GPCR natural variants annotated by Uniprot. According to the GRoSS alignment 1289 of these lie in the TM regions and are listed here with the corresponding BW number. For each mutation we computed its distance to the closest NACHO (or CHICO respectively) residue on the same TM. Zero means this residue is the NACHO (CHICO) residue, in which case we also provide the multiplicity column counting to how many NACHO (CHICO) contacts this residue belongs to. We found 13 (23 including olfactory) mutations of residues on both lists, 48 (99 including olfactory) on the NACHO only list, and 161 (299 including olfactory) on the CHICO only list.

### Molecular graphics

3D molecular views have been rendered using PyMOL [81].

## Results and Discussion

### Gaps in the alignment of TM regions

We constructed the GRoSS alignment in order to avoid gaps in the TM regions and to simplify preparation of homology models. With the BW residues correctly aligned, and without any gaps in the TM regions, we can use this alignment for direct generation of homology models of the TM helix bundle using essentially any structural template.

A general approach to preparing homology models is to create a new alignment for each target and available template, say using the HMM-HMM method [77]. However, HMM-HMM often produces false gaps in the TM regions. Recently, a sequence numbering for GPCR crystal structures was presented by Isberg et al. [24] (available at GPCRDB [78]) that used a structural alignment to identify gaps or bulges in TM regions, when comparing the same TM between any two crystal structures. The properly placed gaps, often improve structural alignment of helix kinks or loose turns. However, the best structural alignment also resulted in gaps in TM regions that can never be predicted by sequence alignment, such as HMM-HMM alone. Table A of S1 Text shows that the mismatches between the GPCRDB and HMM-HMM are common. The GPCRDB alignment is good for retrospective analysis of known structures, but cannot be used for predictions of unobserved gaps.

To quantify the differences between homology models based on the three difference alignments, we compare the RMSD, TM-score and number of common contacts in Fig A of S1 Text. Overall, these comparisons show that GRoSS performs similarly to GPCRDB for alignments within one class, and better for inter-class alignments. GRoSS performs better than HMM-HMM within a class, and significantly better between different classes.

### Loop alignment

We omit loops from the GRoSS alignment, because in the GPCR protein superfamily loops are very diverse, especially the loops EC2 and IC3. EC2 is up to 171 residues long for some class A receptors, but it is shorter than 35 residues in class C, and shorter than 20 residues for all other receptors. IC3 is up to 223 residues long for some class A receptors, but it is shorter than 20 residues for all other receptors. There are likely important similarities among the loops across the GPCR classes. For example, on the intracellular side the receptors have to be sufficiently similar to accommodate G-proteins and arrestins. Furthermore, on the extracellular side, there is a highly conserved disulfide bond between TM3 and loop EC2 that is important for the assembly of the receptor in the membrane [82]. Thus it is possible that with more experimental GPCR structures, a more systematic understanding of the loop regions will emerge as well.

### Sequence alignment from structural alignment

Fig 2 compares class A to the other classes for the alignment constructed by maximizing the number of common inter-helical contacts (Table 2). The purple color in Fig 2 denotes the structural contacts common to all classes, and orange denotes contacts specific to class A. Only one contact, 6.51–7.39, is present in all of class A structures (active and inactive), but it is not in the structures of the other classes. Furthermore, the interactions of TMs 1–5 are more conserved across all classes, but the TM 6 and 7 contacts are more class A specific. It is possible that during the GPCR assembly the helices 1–5 form some intermediate partially folded state before helices 6 and 7 are fully present in the membrane. This might be the reason why the contacts between helices 1–5 are more similar across the classes.

**Fig 5:**
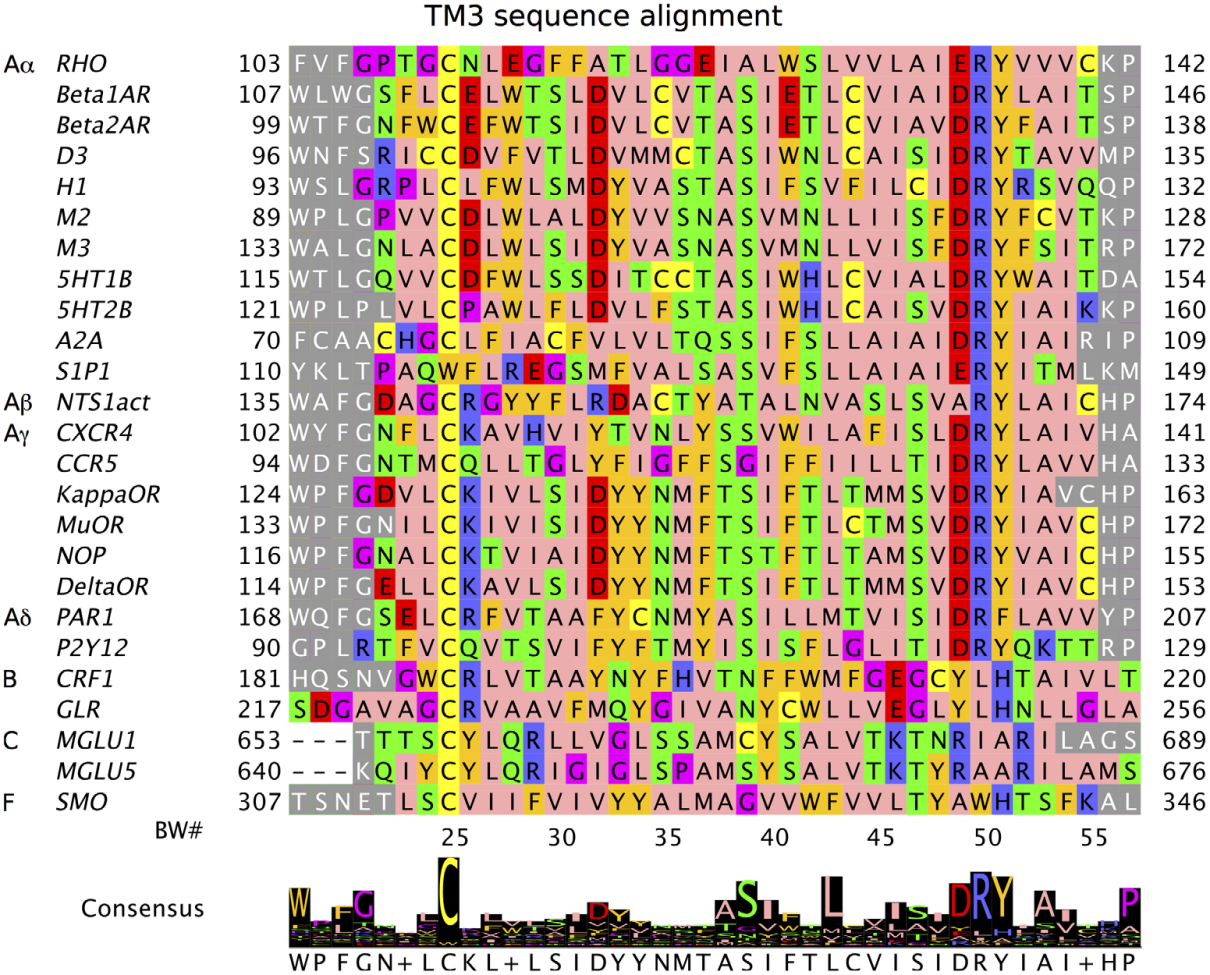
TM 3 sequence alignment for the 25 crystal structures. Other TMs are shown in Fig 6. The sequences are taken from the selected PDB files. The TM helix residues are colored in the Zappos scheme, which captures the chemical nature of each residue (e.g. helix breakers, proline and glycine, are shown in purple). The loop residues are shown in grey. The BW n.50 residue (numbering displayed below the sequences) is the most conserved within the class A. The consensus sequence is most similar to class A, because most sequences are from this class. The largest differences are for the last 5 sequences, which belong to the classes B, C, and F. The figure was prepared using Jalview.

**Fig 6:**
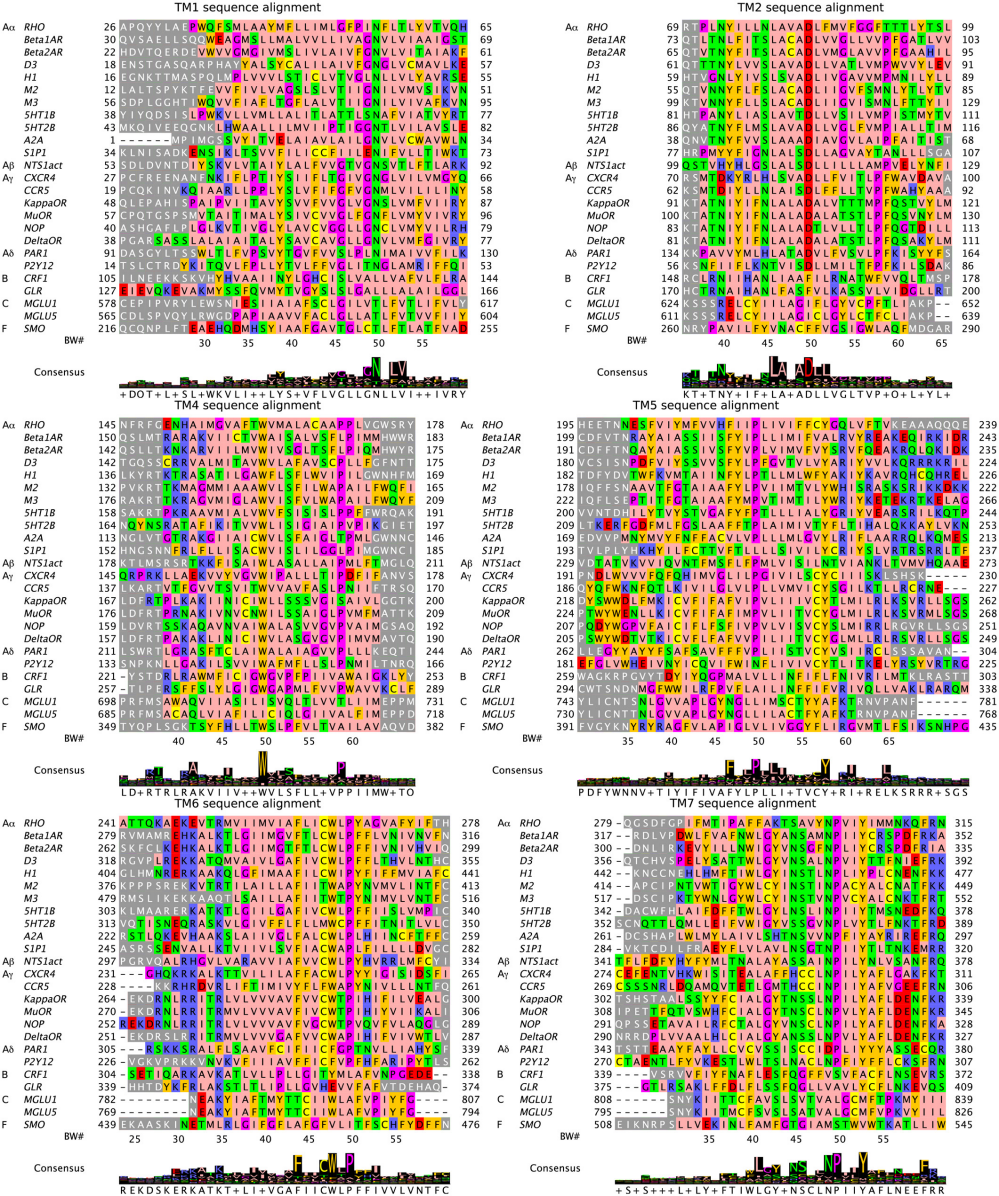
Sequence alignments for TMs 1,2,4–7 for the 25 crystal structures. Same caption as Fig 5, where TM3 is shown.

Fig 5 shows the alignment of the TM3 regions for all the known crystal structures (other TMs are shown in Fig 6). We see that the DRY motif at positions 3.49–3.51 is highly conserved within the 20 class A sequences, and even when there are mutations only similar amino acids occur: ERY, DRF (however, there exist class A GPCRs without this motif, e.g. PTGDR has ECW [83]). In classes B, C, and F the DRY motif is not conserved at all.

Proline residues often cause a helix kink and are commonly found in the TM domains of membrane proteins. They are structurally important for deciding which structures should be used as templates for modeling a new protein. In Fig 5, prolines are highlighted in purple. For example, only MGLU5 has a proline in a central region of TM3, but in this case, the shape of TM3 is very similar to MGLU1, which does not have the corresponding proline.

The consensus sequence for TM3 mostly agrees with class A residues, because most of the crystal structures are from the class A. Interestingly the most conserved residue across all classes is Cys3.25, which forms a disulfide bond to the extracellular loop EC2. This bond is important for the stability of the protein, and shown to be critical for the GPCR assembly [82].

Fig 6 shows the consensus sequence and alignment of the remaining TM regions (1,2,4,5,6,7) for all experimental structures considered. Known conserved residues in these TMs for class A receptors are easily spotted. For TM1, residue 1.50 is a conserved polar residue for all GPCR classes except class B. For TM4, W4.50 residue is conserved across all classes except class C. For TM5, residue 5.60 is a positively charged residue for classes B, C, F, and most A*α* receptors. For TM6, residue W6.48 is conserved for all classes except A*δ*, B, and F. TM7 residue 7.45 is exceptional in being a conserved polar residue across all classes and also appears on our conserved contact residue list (see below).

The conserved inter-helical contacts of class A were the basis for the alignment between the GPCR classes. These contacts show interactions that should be considered first in analysis of the structure or function of these proteins. Fig 2 shows the contacts conserved only within class A, and a similar analysis is shown for classes B, C, and F in S9 Fig. However, since only one or two structures are available in classes B, C, and F, the resulting list is not averaged as it was for class A, and it will be refined as more crystal structures from these classes become available. Thus we cannot yet determine which residues are causing most of the systematic differences between the classes and which residues are critical within each particular class.

### Common contacts between different classes define the GPCR structural fold

There are 40 inter-helical contacts common to class A GPCRs as shown in orange in Fig 2. Out of these, 23 contacts (shown in purple) are present in the crystal structures from classes B, C, and F as well. These 23 conserved inter-helical contacts (CHICOs) formalize our initial insight that the TM bundles of all the different classes are similar and define the GPCR “structural fold”. As more structures become available, this structural fold will be refined.

Examining the inter-helical contacts that make up this GPCR structural fold, we find that TM6 is fully decoupled from both TM3 and TM5, whereas this was not the case for the fold that corresponds to only class A GPCRs (orange contacts in Fig 2). GPCRs across different classes couple to the same set of G proteins and arrestins, and now it is known from overwhelming structural and biophysical evidence that G proteins [6] and arrestins [8] couple to the GPCRs between TM3/TM6 or TM5/TM6 regions. The GPCRs have evolved to conserve these functionally important couplings with their intracellular signal transduction partners, but have not had the need to conserve contacts of TM6 with TM3 or TM5. This is consistent with the structural fold of GPCRs shown by purple contacts in Fig 2.

Fig 2 seems to suggest that there are two separate conserved units: TMs 1–5 and TMs 6–7. It is possible that for receptors that have very long loop IC3 (these are only in class A), the TMs 1–5 need to stabilize in the membrane prior to assembly of the last two TMs.

Another important observation is that the specific conserved contact residues defining the GPCR structural fold are *not* conserved across the different GPCR classes. This tactic of nature to maintain a structural fold without conserving the residues is not uncommon, e.g., the MAT*α*2 homeodomain-operator complex in yeast and drosophila has maintained the homeodomain-fold structure to interact with DNA, even though the species are separated by millions of years and have poor sequence homology in this domain [84].

### Phylogenetic tree

**Fig 7:**
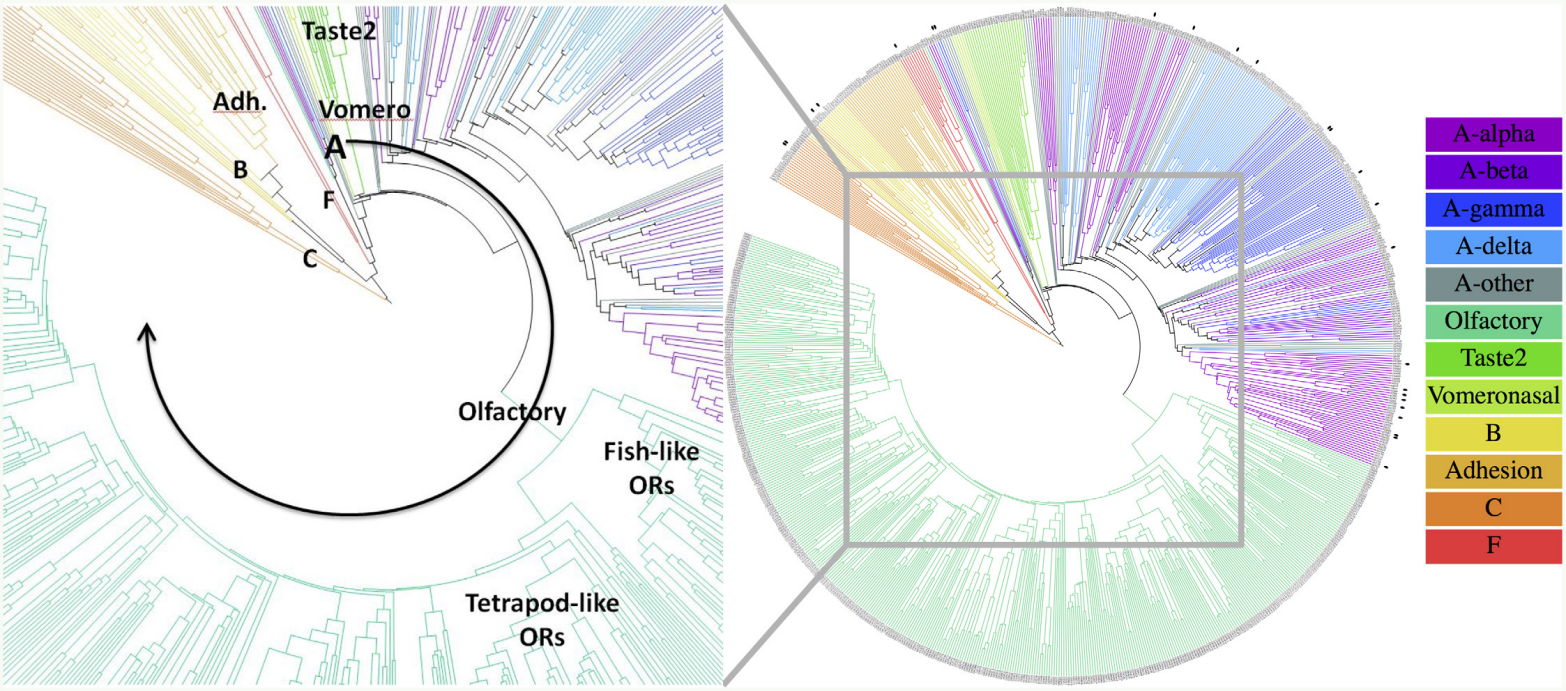
The phylogenetic tree based only on TM similarity using the GRoSS alignment (loops were ignored). Color coding denotes the GPCR class. Proteins with known crystal structure are emphasized with a dot. The full resolution version of this figure is in S4 Fig.

The sequence similarities between the TM regions of the crystal structures are shown in S2 Fig (two residues are considered similar if their BLOSUM62 matrix entry is positive). The similarities are higher than 40% for proteins within the class A branches, from 34% to 54% across the class A branches, and 18–36% across the classes. Based on the GRoSS alignment, we computed the similarity for all the human GPCR proteins. The phylogenetic tree in Fig 7 graphically captures sequence similarity between all the proteins, which also indirectly corresponds to their structural differences (we compare these below). Even though evolutionary considerations were ignored when constructing this tree (for phylogenetic analysis see e.g. the Evolutionary Trace method [85]), this phylogenetic tree clearly contains evolutionary information, but it may miss the information encoded in the loops.

The branches near the root of our tree are very sensible: First class C separates, then class B and adhesion proteins branch off, then class F, and finally class A comprising the rest of the tree. Evolutionarily, this is consistent with the recent most detailed analysis of 83 species [86], which showed that glutamate receptors (class C) and bacterial cAMP receptors are the oldest (>1400 MYA, million years ago), followed by class B and F (∼1275 MYA), and lastly rhodopsin-like receptors (class A)(∼1100 MYA). Except for several outliers, the first major branches to separate in class A are the sensory receptors: Vomeronasal, Taste2, and Olfactory. In the olfactory branch, the first split separates the fish-like receptors (families 51–56) from the tetrapod-like receptors (families 1–13). The subdivision of the rest of the class A does not follow the *α*−*δ* subclasses, but it is close. Near the leaves (i.e., for closely related proteins), the displayed tree might not provide the best classification, since our computation of similarity ignored loops. For related proteins, it may be advantageous to include similarity of the loops as well, since loops often interact with ligands, and therefore can determine receptor specificity. Indeed, it is somewhat surprising that using only the TM domain alignment of all human GPCRs, a phylogenetic tree can be constructed that correctly gets most evolutionary signatures of GPCRs. This suggests that the TM domains of GPCRs contain a good part of the signatures of the divergent evolution of GPCRs, including evolutionary separation of different classes, whose visible differences are usually seen in their soluble domains (N/C-termini and intracellular/extracellular loops).

### Conserved inter-helical contacts involved in activation provide functionally important residues

We found that 40 inter-helical contacts are present in at least 23 out of 24 class A crystal structures (CHICOs in Fig 2). We infer that these residues are important for the interactions between the helices and that any changes to these residues may cause structural stability issues for the protein. Thus, naturally occurring mutations of the residues involved in the conserved contacts could be direct causes of physiological differences and/or diseases.

Comparing the common contacts among different proteins is not straightforward because many of the sequence differences appear random. Focusing on the difference between active and inactive conformations of the same protein makes the significance of the individual residues much clearer. There are 3 active-inactive crystal structure pairs available: RHO, *β*_2_AR, and M2 with accepted “fully active” conformations. The active structure of A2A is only partially active, and for NTS1act the inactive structure is not available.

**Fig 8:**
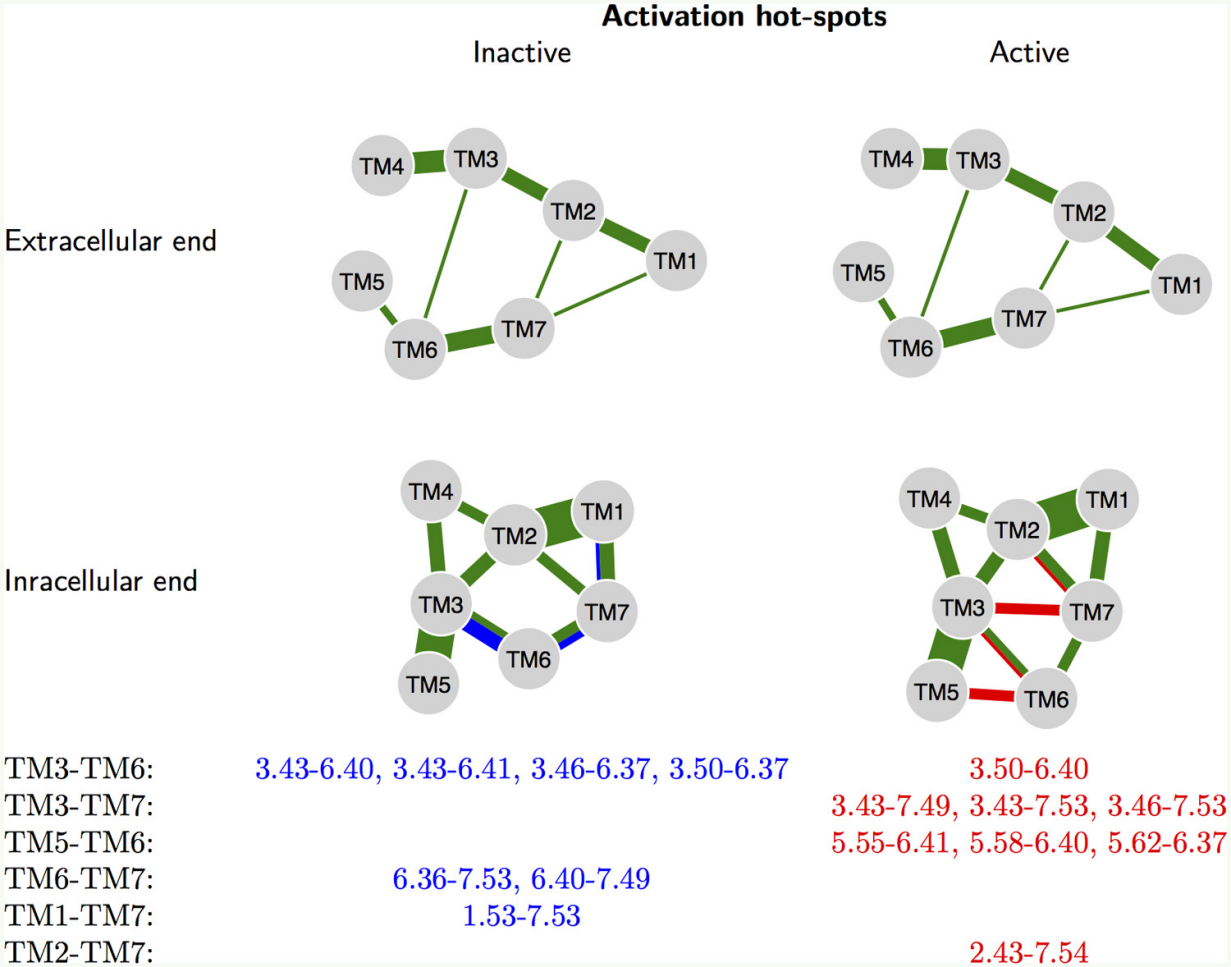
Native activation “hot-spot” residues (NACHOs), which are contacts that change upon receptor activation. The width of the green lines is proportional to the number of contacts common to all six structures (RHO, *β*_2_AR, M2, and their active structures). Blue shows the contacts present only in inactive structures, and not in inactive structures; while red shows the opposite. The upper diagrams show contacts in the extracellular half of the membrane. We see that there is no systematic change common to the class A receptors in the conformation of the extracellular half of the TMs. This is not obvious, because there are conformational changes accompanying ligand binding. All the systematic changes, which enable G protein binding, occur in the intracellular half of the TMs. The list only contains 15 different residues in 15 different contacts. Thus many of the residues switch partners upon activation.

The main signature of activation for rhodopsin is breaking the R3.50↔E6.30 salt-bridge and forming of the K5.66↔E6.30 salt-bridge. Instead of keeping track only of hydrogen bonds, our analysis of contacts allows us to determine more general changes during the activation. The changes in structural contacts upon activation are shown in Fig 8 along with the list of contact residues that change, referred to as native activation “hot-spot” residues (NACHOs).

An important observation is that the structural contacts in the extracellular half of the receptors do not change upon activation. All structural changes occur in the intracellular region, where the contacts get rewired upon the binding of the G protein. Most of the changes occur for TM 6, since the intracellular end of helix 6 undergoes the largest movement upon activation. However, TM 7 also shows a large number of systematic changes, as it breaks a contact with TM1 and creates new contacts with TM 2 and 3. The residues 3.43 and 3.46 occur in the list of conserved contacts in both active and inactive structures, therefore the conformational changes around these residues seem to be very important for the conformational changes during activation. The class A switching mechanism seems to rely critically on a small number of NACHO residues (15 residues). If any of these residues is mutated, the energy landscape of the active and inactive states might be modified, making the receptor likely to become either constitutively active or inactive, thereby altering or breaking its natural function.

### Examples of mutations and natural variants modifying the function

It has been shown experimentally that single amino acid mutations can have a dramatic effect on GPCR activity. For example, the man-made mutation T3.46A makes the receptor CB1 fully inactive, while the mutations T3.46I and L3.43A make it constitutively active [87, 88]. Both positions, 3.46 and 3.43, are on the NACHO list of residues critically involved in activation. These particular mutations were introduced by experimentalists, but the NACHOs (Fig 8) are useful for judging the effect of natural variants as well. The positions of many single nucleotide polymorphisms (SNPs) are known from genetic studies, and by using the global GPCR alignment, we can determine the BW position of each SNP residue. Then each position can be directly compared against the list of activation hot-spots (and to the list of conserved contacts) to estimate the variant’s importance: whether the mutation causes some structural defects or whether it is likely to be benign. We scanned the Uniprot database [31] for naturally occurring mutations for all human GPCRs and converted the residue numbering to the BW scheme using our alignment. Table 3 provides several examples [87–98].

**Table 3:** Examples of natural variants and mutations that are associated with functional change or disease and which coincide with the NACHO residues.

Class	Protein	Uniprot	G-protein	Mutation	BW#	Activity Change	Disease Association	Reference
A-alpha	**CB1**	P21554	Gi/Go, Gs	T210A	**3.46**	Inactive	None	[87]
				T210I	**3.46**	Highly constitutively active	None	[87]
				L207A	**3.43**	Highly constitutively active	None	[88]
A-beta	**V2R**	P30518	Gs	R137C^a^	**3.50**	Constitutively active	NSIAD	[89]
				R137L^a^	**3.50**	Constitutively active	NSIAD	[89]
A-gamma	**CCR5**	P51681	Gi/Go	R126N	**3.50**	Disables G-protein coupling	None	[90]
A-delta	**FSHR**	P23945	Gs	R573C^a^	**6.36**	Reduces AC stimulation	Ovarian dysgenesis 1	[91]
B	**PTH1R**	Q03431	Gs, Gq/G11	T410P^a^	**6.37**	Constitutively active	JMC	[92]
				T410R^a^	**6.37**	Active (less than T410P)	JMC	[93]
				H223R^a^	**2.43**	Constitutively active	JMC	[92]
C	**CASR**	P41180	Gi/Go, Gq/G11, G12/G13	F788C^a^	**5.55**	More active than wild type	Hypocalcemia	[94]
				F806S^a^	**6.36**	No significant activating effect	Hypocalcemia	[95, 96]
				F788L^a^	**5.55**	More active than wild type	Hypocalcemia	[97]
F	**FZD4**	Q9ULV1	G12/G13	K436T^a^	**6.36**	Not known	Colorectal cancer	[98]
**Predictions**								
A-alpha	**DRD5**	P21918	Gs	T297P^a^	**6.36**	Predicted change of function	Not known	
Adhesion	**GPR56**	Q9Y653	Gq/G11, G12/G13	M493T^a^	**3.43**	Predicted change of function	Not known	

^a^ Natural variant.

For example, the natural variants R3.50C and R3.50L cause the vasopressin V2 receptor to be constitutively active. This causes “nephrogenic syndrome of inappropriate antidiuresis”, which presents itself as an inability to excrete a free water load, resulting in low sodium levels [89]. The mutations of R3.50 clearly interfere with arginine’s ability to form hydrogen bonds, and so they disrupt the activation mechanism.

Similarly the natural variant H2.43R in Parathyroid hormone receptor causes its constitutive activity. This mutation of class B receptor causes “Jansen metaphyseal chondrodysplasia”, which is characterized by short-limbed dwarfism [99]. Since the same G proteins couple to different GPCR classes, we can expect the same or similar structural signatures of activation in class B as in class A.

For both of the above examples, the mutations are known to cause constitutive activity. However, there are many observed natural variants, for which the effect is unknown. For example, we predict that the natural variant M3.43T of GPR56 will influence its activation, because the residue 3.43 has to switch contact residues during activation. This adhesion GPCR is involved in cell adhesion as well as in cell to cell interactions, and regulates the migration of neural precursor cells; thus the mutation likely has serious consequences. No databases of single nucleotide polymorphisms contain any functional information about this mutation (we checked Uniprot, and the GPCR specific TinyGRAP [33] and NAVA [32] databases), therefore this is a new prediction based on the analysis of the GPCR fold presented here. Another prediction can be made for the natural variant T6.36P of the D_5_ dopamine receptor. This is a class A receptor and it influences the activity of adenylyl cyclase. Again, we predict that the natural variant T6.36P dramatically changes activation response of this receptor, either to be more constitutively active or less active.

We have illustrated the importance of the NACHO residues by finding disease associations that are caused by single mutations at these positions. The list of NACHO residues only contains 15 residues, which is about 5% of the transmembrane domain. Similarly, we hypothesize that mutations at the CHICO positions (that define the structural fold) can dramatically change the receptor function. There are many known natural variants whose effect has not been experimentally studied yet, and these criteria can be used to focus (experimental) attention on variants, which cause dramatic changes.

From Uniprot, we collected all 2449 GPCR natural variants, of which about half (1289) lie in the TM regions. These are listed in S3 Table together with their functional or disease associations, if available on Uniprot. Table 4 summarizes the limited disease-association data available for mutations in GPCRs. It shows that about half (∼53%) of the GPCR TM residue mutations have been found to be associated with diseases. This number jumps to about two-thirds for CHICO or NACHO residues (∼67% and ∼66% respectively) and almost all (12 out of 13 or ∼92%) for residues that appear on both CHICO and NACHO lists. This strongly suggests that NACHO and CHICO residues can help prioritize mutation sites to guide experimental validation of the structural and functional hypotheses presented by these specific residues.

**Table 4:** Summary of SNPs annotated on Uniprot. The complete list is in S3 Table.

	Number of SNPs	With disease annotation	% with disease
All GPCRs	2489	694	27.9
All TM regions	1289	363	28.2
Excluding olfactory and unassigned	1463	635	43.4
TM	652	346	**53.1**
Non TM (Nterm+loops+Cterm)	811	289	35.6
CHICO only	161	105	65.2
CHICO	174	117	**67.2**
NACHO only	48	28	58.3
NACHO	61	40	**65.6**
Both CHICO and NACHO	13	12	**92.3**

There are still many SNPs in S3 Table that have unknown functional implications. We sort them with respect to a score capturing their relative position to the CHICO and NACHO residues: “distance to the closest NACHO + distance to CHICO - multiplicity of the closest NACHO - multiplicity of CHICO + Blosum62 of the mutation”. We hypothesize that the entries with the lowest score are very likely to cause dramatic changes in the receptor structure and function. The full list thus provides a large number of testable hypotheses about the molecular basis of disease-associated SNPs.

The CHICO and NACHO residues are results of a structural comparison, but the functional relevance of mutations is often obtained from phylogenetic considerations instead of structural ones. In S4 Table we compare these two approaches. We consider variations in sequences among a curated list of 77 P2Y12 orthologs [100], and among orthologs in an uncurated database for multiple proteins [101]. The CHICO and NACHO positions are more conserved than other TM residues in all GPCR classes among orthologs; and residues present on both lists are even more conserved. Thus both approaches are consistent, and should be combined to form more detailed insights.

### Size of helix movement in available crystal structures and implications for homology modeling

By analysis of the inter-helical contacts we constructed the GRoSS alignment between all the GPCR proteins, from which new homology models can be derived. For structure prediction we would like to know how far the homology models are from the target structure. The variability of the TM bundle can be measured using the available crystal structures.

**Fig 9:**
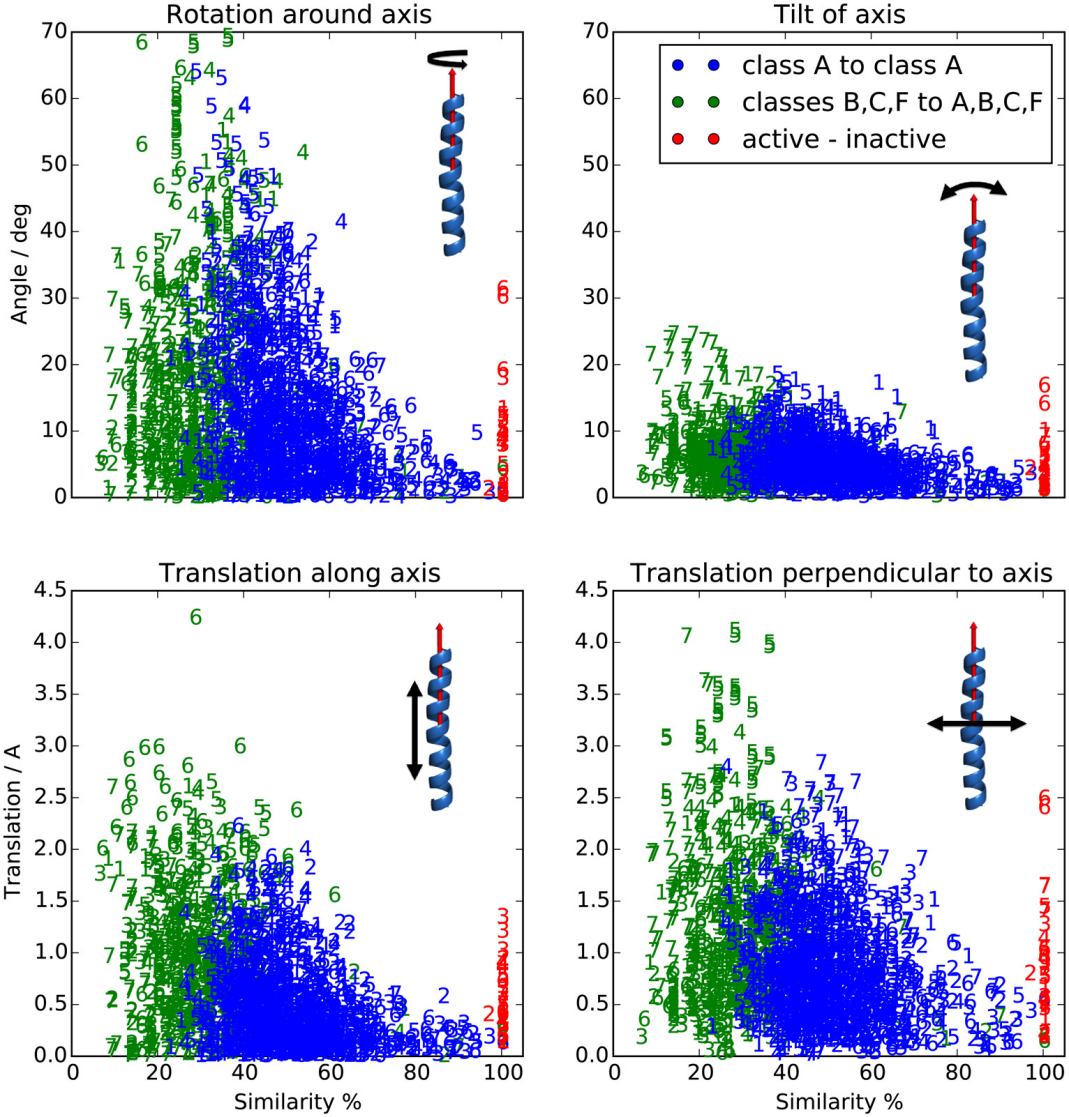
Magnitude of the rigid body moves of the helices necessary to map one structure to another. All TMs 1–7 from all available structure pairs were compared and each symbol denotes which TM is the data point from. The coordinate system is defined in the text. The maximal observed deviation is approximately proportional to the sequence dissimilarity of the two compared TMs, and it follows the same trend within class A (blue symbols) and across the GPCR superfamily (green symbols). The red symbols, which correspond to the active-inactive structure pairs, show rigid body moves caused by receptor activation. S10 Fig has an analogous plot of residual RMSD vs. similarity for each helix after the best rigid body transformation. RMSD shows a similar trend as the plots in this figure.

Fig 9 shows the observed move sizes, when the individual TM helices are treated as rigid bodies. Each pair of known structures was first aligned together, then each helix of the first protein was individually aligned to the corresponding helix of the second protein and the size of the move was measured. The center of mass translation was broken down into the direction along the helical axis and a direction perpendicular to it. The “tilt of axis” measures how much axis 1 had to be rotated to axis 2. And finally the “rotation around axis” measures the necessary rotation around the axis to map the corresponding atoms to each other.

The maximal move sizes that need to be considered get smaller as the similarity of the TM sequence increases. If we are predicting a structure starting from a homology model with higher than 50% similarity, then we need only consider translating the helices up to 1.5 Å in any direction, tilting them up to 10°, and rotating around their axis by 40°. This is a very useful bound for refining homology models.

The same comparison can be applied to a single protein in multiple conformations. The red points in Fig 9 show the magnitude of rigid body moves undergone during activation for the 3 available pairs of active-inactive structures. Activation involves mainly the movement of TMs 5, 6, and 7. The computation of the move sizes ignores the bending of TM6 during activation, so it should be understood as an approximate description only.

### Conclusion

A conceptual understanding of the molecular mechanisms behind biased signaling and functional selectivity is emerging around the conformational flexibility and dynamics of GPCRs. This is supported by an ever-increasing number of experimental and computational studies [102–107] that point to different ensembles of receptor conformations behind the pleiotropic signaling of GPCRs. These mechanistic studies have gotten a significant boost from the recent dramatic developments in GPCR structure determination methods.

We constructed the GRoSS sequence alignment of the transmembrane regions for all known human GPCRs. Although the inter-helical contact residue correspondence in the GRoSS alignment is in many cases approximate (intra-class) or non-existent (inter-class), several inter-helical contacts are highly conserved across the classes, which suggests their importance in the evolutionarily conserved GPCR fold. Our conserved contact analysis of the experimentally observed inactive and active conformations of Rhodopsin, Muscarinic M2, and adrenergic *β*_2_AR identified 15 residue positions in the TM regions that change molecular contacts upon activation. Targeted or natural mutations of these residues are known to cause dramatic changes in the receptor signaling. We recommend that these be the starting point for examining mechanisms for activation and for deleterious mutations. The GRoSS alignment of the TM domains also leads to a functional phylogenetic tree that captures many evolutionary signatures of GPCR evolution. This shows that the class-level differences in the GPCR superfamily are encoded in the TM domains even though the divergence in the loop domains is usually used to distinguish the classes from each other.

The GRoSS alignment is also a promising starting point for structure prediction, as there are no gaps present in the TM domains. For a protein in question one can build homology models based on any of the available templates by mutating the corresponding amino acids. While the increasing coverage of proteins by the crystal structures makes it easier to find a close template, the GRoSS alignment allows remote homology modeling, which can be particularly useful for modeling active states for all GPCR classes. GPCRs are too large (>300 residues) for exploring larger conformational changes only using molecular dynamics. Comparing the available structures with respect to the GRoSS alignment gives approximate bounds on the size of rigid body moves needed for the TM helices to reach the target structure.

The GRoSS alignment is unique in aligning all human GPCR sequences by maximizing the number of conserved inter-helical contacts. These conserved contacts provide a basis for defining a GPCR superfamily-wide structural fold, functionally conserved residue positions (even if residue type may not be conserved), and activation hot-spot residues (NACHOs).

## Supporting Information

S1 Table List of studied GPCR crystal structures. When multiple structures are available, then the one with the highest resolution or the one with least deformed TM helices is used.
(PDF)

S2 Table GRoSS sequence alignment for all 817 human GPCRs. S1 File has this alignment in fasta format. Since there are no gaps in the TM domains, the alignment of each protein is uniquely determined by the BW.50 residues for each TM 1 through 7. We list also the expected range of the helical TM regions, which is estimated as the average TM region in the known crystal structures from the same class. In the discussion of the bitter taste receptors (TAS2Rs), we identified two possible alignments of TM6, but only the first one is presented in the following table. The second choice is to decrease the start, end, and BW50 residue of TM6 by 4.
(CSV)

S3 Table GPCR natural variants annotated by Uniprot mapped to BW numbering and indicating their proximity to the NACHO and CHICO residues. The mutations are ordered according to the following score: “distance to the closest NACHO + distance to CHICO - multiplicity of the closest NACHO - multiplicity of CHICO + Blosum62 of the mutation”.
(CSV)

S4 Table Conservation of CHICO and NACHO residues among orthologs. For orthologs of several proteins we computed average amino acids conservation over TM, and over CHICO/NACHO residues. The data shows that CHICO and NACHO positions are more conserved than other TM residues in all GPCR classes. Residues present on both lists are even more conserved. Two measures of conservation provided by Jalview are used: Consensus is the percentage of orthologs sharing the human amino acid; and Conservation is a qualitative measure counting the number of conserved chemical properties. For P2Y12, we used a curated list of 77 orthologs from [100]. For other proteins, we collected predicted orthologs from the MetaPhOrs database (release 201405 [101]), aligned them with Clustal Omega, and then removed sequences with gaps in the TM regions.
(PDF)

S1 Fig Detailed view of conserved motifs in class A GPCRs. The conserved residues in 24 different structures (including active) have very similar positions, which shows that the class A GPCR fold is highly conserved. The full TM bundle is shown in Fig 1.
(PDF)

S2 Fig Sequence similarity (%) of the TM bundles between crystal structures for the final sequence alignment. Two residues are similar if their BLOSUM62 entry is positive.
(PDF)

S3 Fig Backbone (atoms N, C_*α*_, C, O) RMSD of the TM bundles for the final sequence alignment. For a given pair of structures, there may exist a different sequence alignment, which results in a lower RMSD than the listed one.
(PDF)

S4 Fig High-resolution phylogenetic tree (Fig 7) based on TM similarity only. The pdf file is searchable for the UNIPROT accession numbers. Loops were ignored. Color coding denotes the GPCR class. Proteins with known crystal structure are emphasized with a dot.
(PDF)

S5 Fig Testing the robustness of the alignment of the Vomeronasal receptors with the other groups. This is an extended version of Fig 3, same caption.
(PDF)

S6 Fig Testing the robustness of the alignment of the Taste2 receptors with the other groups. This is an extended version of Fig 4, same caption.
(PDF)

S7 Fig Testing the robustness of the alignment of the Vomeronasal receptors with the GPCRtm substitution matrix. Same caption as in Fig 3.
(PDF)

S8 Fig Testing the robustness of the alignment of the Taste2 receptors with the GPCRtm substitution matrix. Same caption as in Fig 4.
(PDF)

S9 Fig Diagram of interhelical contacts present in classes B, C, and F. The width of the line connecting two TMs is proportional to the number of contacts present in all structures from the given class. The list in red font shows the contacts not present in any available structure from other classes.
(PDF)

S10 Fig RMSD of helices after best rigid body move. Same caption as Fig 9.
(PDF)

S1 Text Comparison of the GRoSS alignment to the HMM-HMM alignment [77] and to the GPCRDB alignment [24, 78]. (DOCX)

S1 File The GRoSS alignment in fasta format and annotation of the TM regions and BW residues in Jalview format. The first 29 sequences are the actual sequences from the PDB files of used crystal structures; the rest of the sequences are from Uniprot. N-terminal, loops and C-terminal are not aligned. For interactive work it is useful to also highlight the TM regions and BW residues using the Jalview annotation gross-alignment.gff file.
(ZIP)

## References

[1] Lefkowitz RJ. Historical review: a brief history and personal retrospective of seven-transmembrane receptors. Trends Pharmacol Sci. 2004 8;25(8):413–422. 10.1016/j.tips.2004.06.006 15276710

[2] Hill SJ. G-protein-coupled receptors: past, present and future. Br J Pharmacol. 2006 1;147 Suppl 1:27–37. 10.1038/sj.bjp.0706455 PMC1760739 16402114

[3] Zalewska M, Siara M, Sajewicz W. G protein-coupled receptors: abnormalities in signal transmission, disease states and pharmacotherapy. Acta Pol Pharm. 2014;71(2):229–243. 25272642

[4] Garland SL. Are GPCRs Still a Source of New Targets? Journal of Biomolecular Screening. 2013;18:947–966. 10.1177/1087057113498418 23945874

[5] Stevens RC, Cherezov V, Katritch V, Abagyan R, Kuhn P, Rosen H, et al The GPCR Network: a large-scale collaboration to determine human GPCR structure and function. Nat Rev Drug Discov. 2013 1;12(1):25–34. 10.1038/nrd3859 23237917 PMC3723354

[6] Rasmussen SG, DeVree BT, Zou Y, Kruse AC, Chung KY, Kobilka TS, et al Crystal structure of the *β*2 adrenergic receptor-Gs protein complex. Nature. 2011 9;477(7366):549–555. 10.1038/nature10361 21772288 PMC3184188

[7] Chung KY, Rasmussen SG, Liu T, Li S, DeVree BT, Chae PS, et al Conformational changes in the G protein Gs induced by the *β*2 adrenergic receptor. Nature. 2011 9;477(7366):611–615. 10.1038/nature10488 21956331 PMC3448949

[8] Kang Y, Zhou XE, Gao X, He Y, Liu W, Ishchenko A, et al Crystal structure of rhodopsin bound to arrestin by femtosecond X-ray laser. Nature. 2015 7;523(7562):561–567. 10.1038/nature14656 26200343 PMC4521999

[9] Rosenbaum DM, Rasmussen SG, Kobilka BK. The structure and function of G-protein-coupled receptors. Nature. 2009 5;459(7245):356–363. 10.1038/nature08144 19458711 PMC3967846

[10] Hanson MA, Stevens RC. Discovery of new GPCR biology: one receptor structure at a time. Structure. 2009 1;17(1):8–14. 10.1016/j.str.2008.12.003 19141277 PMC2813843

[11] Heifetz A, Schertler GF, Seifert R, Tate CG, Sexton PM, Gurevich VV, et al. GPCR structure, function, drug discovery and crystallography: report from Academia-Industry International Conference (UK Royal Society) Chicheley Hall, 1–2 September 2014. Naunyn Schmiedebergs Arch Pharmacol. 2015 Mar;388:883–903. 10.1007/s00210-015-1111-8 PMC4495723 25772061

[12] Ballesteros JA, Weinstein H. Integrated methods for the construction of three-dimensional models and computational probing of structure-function relations in G protein-coupled receptors In: Sealfon SC, editor. Receptor Molecular Biology. vol. 25 of Methods in Neurosciences. Academic Press; 1995 p. 366–428.

[13] Visiers I, Ballesteros JA, Weinstein H. Three-dimensional representations of G protein-coupled receptor structures and mechanisms. Meth Enzymol. 2002;343:329–371. 10.1016/S0076-6879(02)43145-X 11665578

[14] Venkatakrishnan AJ, Flock T, Prado DE, Oates ME, Gough J, Madan Babu M. Structured and disordered facets of the GPCR fold. Curr Opin Struct Biol. 2014 8;27:129–137. 10.1016/j.sbi.2014.08.002 25198166

[15] Moult J, Fidelis K, Kryshtafovych A, Schwede T, Tramontano A. Critical assessment of methods of protein structure prediction (CASP)—round X. Proteins: Structure, Function, and Bioinformatics. 2014;82:1–6. 10.1002/prot.24452 PMC4394854 24344053

[16] Barth P, Wallner B, Baker D. Prediction of membrane protein structures with complex topologies using limited constraints. Proceedings of the National Academy of Sciences. 2009;106(5):1409–1414. 10.1073/pnas.0808323106 PMC2635801 19190187

[17] Yarov-Yarovoy V, Schonbrun J, Baker D. Multipass membrane protein structure prediction using Rosetta. Proteins. 2006 3;62(4):1010–1025. 10.1002/prot.20817 16372357 PMC1479309

[18] Michino M, Abola E, Brooks CL, Dixon JS, Moult J, Stevens RC. Community-wide assessment of GPCR structure modelling and ligand docking: GPCR Dock 2008. Nat Rev Drug Discov. 2009 6;8(6):455–463. 10.1038/nrd2877 19461661 PMC2728591

[19] Kufareva I, Rueda M, Katritch V, Stevens RC, Abagyan R. Status of GPCR modeling and docking as reflected by community-wide GPCR Dock 2010 assessment. Structure. 2011 8;19(8):1108–1126. 10.1016/j.str.2011.05.012 21827947 PMC3154726

[20] Kufareva I, Katritch V, Stevens R, Abagyan R. Advances in GPCR Modeling Evaluated by the GPCR Dock 2013 Assessment: Meeting New Challenges. Structure. 2014;22(8):1120–1139. 10.1016/j.str.2014.06.012 25066135 PMC4126895

[21] Latek D, Pasznik P, Carlomagno T, Filipek S. Towards Improved Quality of GPCR Models by Usage of Multiple Templates and Profile-Profile Comparison. PLoS ONE. 2013 02;8(2):e56742 10.1371/journal.pone.0056742 23468878 PMC3585245

[22] Worth CL, Kleinau G, Krause G. Comparative sequence and structural analyses of G-protein-coupled receptor crystal structures and implications for molecular models. PLoS ONE. 2009;4(9):e7011 10.1371/journal.pone.0007011 19756152 PMC2738427

[23] Worth CL, Kreuchwig A, Kleinau G, Krause G. GPCR-SSFE: a comprehensive database of G-protein-coupled receptor template predictions and homology models. BMC Bioinformatics. 2011;12:185 10.1186/1471-2105-12-185 21605354 PMC3113946

[24] Isberg V, de Graaf C, Bortolato A, Cherezov V, Katritch V, Marshall FH, et al Generic GPCR residue numbers—aligning topology maps while minding the gaps. Trends Pharmacol Sci. 2015 1;36(1):22–31. 10.1016/j.tips.2014.11.001 25541108 PMC4408928

[25] Abrol R, Kim SK, Bray JK, Griffith AR, G WA III. Characterizing and predicting the functional and conformational diversity of seven-transmembrane proteins. Methods. 2011;55(4):405–414. Membrane Protein Technologies for Structural Biology. 10.1016/j.ymeth.2011.12.005 22197575 PMC3286597

[26] Dalton JA, Lans I, Giraldo J. Quantifying conformational changes in GPCRs: glimpse of a common functional mechanism. BMC Bioinformatics. 2015;16:124 10.1186/s12859-015-0567-3 25902715 PMC4422131

[27] Bray JK, Abrol R, Goddard WA, Trzaskowski B, Scott CE. SuperBiHelix method for predicting the pleiotropic ensemble of G-protein-coupled receptor conformations. Proc Natl Acad Sci USA. 2014 1;111(1):E72–78. 10.1073/pnas.1321233111 24344284 PMC3890820

[28] Venkatakrishnan A, Deupi X, Lebon G, Tate CG, Schertler GF, Babu MM. Molecular signatures of G-protein-coupled receptors. Nature. 2013;494(7436):185–194. 10.1038/nature11896 23407534

[29] Isom DG, Dohlman HG. Buried ionizable networks are an ancient hallmark of G protein-coupled receptor activation. Proc Natl Acad Sci USA. 2015 5;112(18):5702–5707. 10.1073/pnas.1417888112 25902551 PMC4426463

[30] Wolf S, Grunewald S. Sequence, structure and ligand binding evolution of rhodopsin-like G protein-coupled receptors: a crystal structure-based phylogenetic analysis. PLoS ONE. 2015;10(4):e0123533 10.1371/journal.pone.0123533 25881057 PMC4399913

[31] Bateman A, Martin MJ, O’Donovan C, Magrane M, Apweiler R, Alpi E, et al UniProt: a hub for protein information. Nucleic Acids Res. 2015 1;43(Database issue):D204–212. 25348405 10.1093/nar/gku989 PMC4384041

[32] Kazius J, Wurdinger K, van Iterson M, Kok J, Back T, Ijzerman AP. GPCR NaVa database: natural variants in human G protein-coupled receptors. Hum Mutat. 2008 1;29(1):39–44. 10.1002/humu.20638 17924574

[33] Edvardsen O, Reiersen AL, Beukers MW, Kristiansen K. tGRAP, the G-protein coupled receptors mutant database. Nucleic Acids Res. 2002 1;30(1):361–363. 10.1093/nar/30.1.361 11752337 PMC99129

[34] Fredriksson R, Lagerstrom MC, Lundin LG, Schioth HB. The G-protein-coupled receptors in the human genome form five main families. Phylogenetic analysis, paralogon groups, and fingerprints.Mol Pharmacol. 2003 6;63(6):1256–1272. 10.1124/mol.63.6.1256 12761335

[35] Schioth HB, Fredriksson R. The GRAFS classification system of G-protein coupled receptors in comparative perspective. Gen Comp Endocrinol. 2005 5;142(1–2):94–101. 10.1016/j.ygcen.2004.12.018 15862553

[36] Sharman JL, Benson HE, Pawson AJ, Lukito V, Mpamhanga CP, Bombail V, et al IUPHAR-DB: updated database content and new features. Nucleic Acids Research. 2013;41(D1):D1083–D1088. 10.1093/nar/gks960 23087376 PMC3531077

[37] Sievers F, Wilm A, Dineen D, Gibson TJ, Karplus K, Li W, et al Fast, scalable generation of high-quality protein multiple sequence alignments using Clustal Omega. Molecular Systems Biology. 2011;7(1). 10.1038/msb.2011.75 21988835 PMC3261699

[38] Li J, Edwards PC, Burghammer M, Villa C, Schertler GFX. Structure of Bovine Rhodopsin in a Trigonal Crystal Form. Journal of Molecular Biology. 2004;343(5):1409–1438. 10.1016/j.jmb.2004.08.090 15491621

[39] Choe HW, Kim YJ, Park JH, Morizumi T, Pai EF, Krauss N, et al Crystal structure of metarhodopsin II. Nature. 2011;471(7340):651–5. 10.1038/nature09789 21389988

[40] Cherezov V, Rosenbaum DM, Hanson MA, Rasmussen SG, Thian FS, Kobilka TS, et al High-resolution crystal structure of an engineered human beta2-adrenergic G protein-coupled receptor. Science (New York, NY). 2007;318(5854):1258–65. 10.1126/science.1150577 PMC2583103 17962520

[41] Haga K, Kruse AC, Asada H, Yurugi-Kobayashi T, Shiroishi M, Zhang C, et al Structure of the human M2 muscarinic acetylcholine receptor bound to an antagonist. Nature. 2012;482(7386):547–51. 10.1038/nature10753 22278061 PMC3345277

[42] Kruse AC, Ring AM, Manglik A, Hu J, Hu K, Eitel K, et al Activation and allosteric modulation of a muscarinic acetylcholine receptor. Nature. 2013 12;504(7478):101–106. 10.1038/nature12735 24256733 PMC4020789

[43] Jaakola VP, Griffith MT, Hanson MA, Cherezov V, Chien EY, Lane JR, et al The 2.6 angstrom crystal structure of a human A2A adenosine receptor bound to an antagonist. Science (New York, NY). 2008;322(5905):1211–7. 10.1126/science.1164772 PMC2586971 18832607

[44] Xu F, Wu H, Katritch V, Han GW, Jacobson KA, Gao ZG, et al Structure of an agonist-bound human A2A adenosine receptor. Science (New York, NY). 2011;332(6027):322–7. 10.1126/science.1202793 PMC3086811 21393508

[45] White JF, Noinaj N, Shibata Y, Love J, Kloss B, Xu F, et al Structure of the agonist-bound neurotensin receptor. Nature. 2012;490(7421):508–13. 10.1038/nature11558 23051748 PMC3482300

[46] Warne T, Serrano-Vega MJ, Baker JG, Moukhametzianov R, Edwards PC, Henderson R, et al Structure of a beta1-adrenergic G-protein-coupled receptor. Nature. 2008;454(7203):486–91. 10.1038/nature07101 18594507 PMC2923055

[47] Chien EY, Liu W, Zhao Q, Katritch V, Han GW, Hanson MA, et al Structure of the human dopamine D3 receptor in complex with a D2/D3 selective antagonist. Science (New York, NY). 2010;330(6007):1091–5. 10.1126/science.1197410 PMC3058422 21097933

[48] Shimamura T, Shiroishi M, Weyand S, Tsujimoto H, Winter G, Katritch V, et al Structure of the human histamine H1 receptor complex with doxepin. Nature. 2011;475(7354):65–70. 10.1038/nature10236 21697825 PMC3131495

[49] Kruse AC, Hu J, Pan AC, Arlow DH, Rosenbaum DM, Rosemond E, et al Structure and dynamics of the M3 muscarinic acetylcholine receptor. Nature. 2012;482(7386):552–6. 10.1038/nature10867 22358844 PMC3529910

[50] Wang C, Jiang Y, Ma J, Wu H, Wacker D, Katritch V, et al Structural basis for molecular recognition at serotonin receptors. Science (New York, NY). 2013;340(6132):610–4. 10.1126/science.1232807 PMC3644373 23519210

[51] Liu W, Wacker D, Gati C, Han GW, James D, Wang D, et al Serial femtosecond crystallography of G protein-coupled receptors. Science (New York, NY). 2013;342(6165):1521–4. 10.1126/science.1244142 PMC3902108 24357322

[52] Hanson MA, Roth CB, Jo E, Griffith MT, Scott FL, Reinhart G, et al Crystal structure of a lipid G protein-coupled receptor. Science (New York, NY). 2012;335(6070):851–5. 10.1126/science.1215904 PMC3338336 22344443

[53] Wu B, Chien EY, Mol CD, Fenalti G, Liu W, Katritch V, et al Structures of the CXCR4 chemokine GPCR with small-molecule and cyclic peptide antagonists. Science (New York, NY). 2010;330(6007):1066–71. 10.1126/science.1194396 PMC3074590 20929726

[54] Tan Q, Zhu Y, Li J, Chen Z, Han GW, Kufareva I, et al Structure of the CCR5 chemokine receptor-HIV entry inhibitor maraviroc complex. Science (New York, NY). 2013;341(6152):1387–90. 10.1126/science.1241475 PMC3819204 24030490

[55] Wu H, Wacker D, Mileni M, Katritch V, Han GW, Vardy E, et al Structure of the human *κ*-opioid receptor in complex with JDTic. Nature. 2012;485(7398):327–32. 10.1038/nature10939 22437504 PMC3356457

[56] Manglik A, Kruse AC, Kobilka TS, Thian FS, Mathiesen JM, Sunahara RK, et al Crystal structure of the *μ*-opioid receptor bound to a morphinan antagonist. Nature. 2012;485(7398):321–6. 10.1038/nature10954 22437502 PMC3523197

[57] Thompson AA, Liu W, Chun E, Katritch V, Wu H, Vardy E, et al Structure of the nociceptin/orphanin FQ receptor in complex with a peptide mimetic. Nature. 2012;485(7398):395–9. 10.1038/nature11085 22596163 PMC3356928

[58] Granier S, Manglik A, Kruse AC, Kobilka TS, Thian FS, Weis WI, et al Structure of the *δ*-opioid receptor bound to naltrindole. Nature. 2012;485(7398):400–4. 10.1038/nature11111 22596164 PMC3523198

[59] Zhang C, Srinivasan Y, Arlow DH, Fung JJ, Palmer D, Zheng Y, et al High-resolution crystal structure of human protease-activated receptor 1. Nature. 2012;492(7429):387–92. 10.1038/nature11701 23222541 PMC3531875

[60] Zhang K, Zhang J, Gao ZG, Zhang D, Zhu L, Han GW, et al Structure of the human P2Y12 receptor in complex with an antithrombotic drug. Nature. 2014;509(7498):115–8. 10.1038/nature13083 24670650 PMC4174307

[61] Hollenstein K, Kean J, Bortolato A, Cheng RK, Dore AS, Jazayeri A, et al Structure of class B GPCR corticotropin-releasing factor receptor 1. Nature. 2013;499(7459):438–43. 10.1038/nature12357 23863939

[62] Siu FY, He M, de Graaf C, Han GW, Yang D, Zhang Z, et al Structure of the human glucagon class B G-protein-coupled receptor. Nature. 2013;499(7459):444–9. 10.1038/nature12393 23863937 PMC3820480

[63] Wu H, Wang C, Gregory KJ, Han GW, Cho HP, Xia Y, et al Structure of a class C GPCR metabotropic glutamate receptor 1 bound to an allosteric modulator. Science (New York, NY). 2014;344(6179):58–64. 10.1126/science.1249489 PMC3991565 24603153

[64] Dore AS, Okrasa K, Patel JC, Serrano-Vega M, Bennett K, Cooke RM, et al Structure of class C GPCR metabotropic glutamate receptor 5 transmembrane domain. Nature. 2014 7;511(7511):557–562. 10.1038/nature13396 25042998

[65] Wang C, Wu H, Katritch V, Han GW, Huang XP, Liu W, et al Structure of the human smoothened receptor bound to an antitumour agent. Nature. 2013;497(7449):338–43. 10.1038/nature12167 23636324 PMC3657389

[66] Wang C, Wu H, Evron T, Vardy E, Han GW, Huang XP, et al Structural basis for Smoothened receptor modulation and chemoresistance to anticancer drugs. Nat Commun. 2014;5:4355 10.1038/ncomms5355 25008467 PMC4198951

[67] Lomize MA, Lomize AL, Pogozheva ID, Mosberg HI. OPM: orientations of proteins in membranes database. Bioinformatics. 2006 3;22(5):623–625. 10.1093/bioinformatics/btk023 16397007

[68] Joosten RP, te Beek TA, Krieger E, Hekkelman ML, Hooft RW, Schneider R, et al A series of PDB related databases for everyday needs. Nucleic Acids Res. 2011 1;39(Database issue):D411–419. 10.1093/nar/gkq1105 21071423 PMC3013697

[69] ICM. Manual v.3.0; MolSoft, La Jolla, California, 2012.

[70] Krissinel E, Henrick K. Secondary-structure matching (SSM), a new tool for fast protein structure alignment in three dimensions. Acta Crystallogr D Biol Crystallogr. 2004 12;60(Pt 12 Pt 1):2256–2268. 10.1107/S0907444904026460 15572779

[71] Wootten D, Simms J, Miller LJ, Christopoulos A, Sexton PM. Polar transmembrane interactions drive formation of ligand-specific and signal pathway-biased family B G protein-coupled receptor conformations. Proceedings of the National Academy of Sciences. 2013;110(13):5211–5216. 10.1073/pnas.1221585110 PMC3612682 23479653

[72] Pin JP, Galvez T, Prezeau L. Evolution, structure, and activation mechanism of family 3/C G-protein-coupled receptors. Pharmacol Ther. 2003 6;98(3):325–354. 10.1016/S0163-7258(03)00038-X 12782243

[73] Henikoff S, Henikoff JG. Amino acid substitution matrices from protein blocks. Proc Natl Acad Sci USA. 1992 11;89(22):10915–10919. 10.1073/pnas.89.22.10915 1438297 PMC50453

[74] Rios S, Fernandez MF, Caltabiano G, Campillo M, Pardo L, Gonzalez A. GPCRtm: An amino acid substitution matrix for the transmembrane region of class A G Protein-Coupled Receptors. BMC Bioinformatics. 2015;16:206 10.1186/s12859-015-0639-4 26134144 PMC4489126

[75] Krogh A, Larsson B, von Heijne G, Sonnhammer EL. Predicting transmembrane protein topology with a hidden Markov model: application to complete genomes. J Mol Biol. 2001 1;305(3):567–580. 10.1006/jmbi.2000.4315 11152613

[76] Waterhouse AM, Procter JB, Martin DMA, Clamp M, Barton GJ. Jalview Version 2—a multiple sequence alignment editor and analysis workbench. Bioinformatics. 2009;25(9):1189–1191. 10.1093/bioinformatics/btp033 19151095 PMC2672624

[77] Remmert M, Biegert A, Hauser A, Soding J. HHblits: lightning-fast iterative protein sequence searching by HMM-HMM alignment. Nat Methods. 2012 2;9(2):173–175. 10.1038/nmeth.1818 22198341

[78] Isberg V, Vroling B, van der Kant R, Li K, Vriend G, Gloriam D. GPCRDB: an information system for G protein-coupled receptors. Nucleic Acids Res. 2014 1;42(Database issue):D422–425. 10.1093/nar/gkt1255 24304901 PMC3965068

[79] Singh N, Pydi SP, Upadhyaya J, Chelikani P. Structural basis of activation of bitter taste receptor T2R1 and comparison with Class A G-protein-coupled receptors (GPCRs). J Biol Chem. 2011 10;286(41):36032–36041. 10.1074/jbc.M111.246983 21852241 PMC3195589

[80] Letunic I, Bork P. Interactive Tree Of Life v2: online annotation and display of phylogenetic trees made easy. Nucleic Acids Research. 2011;39(suppl 2):W475–W478. 10.1093/nar/gkr201 21470960 PMC3125724

[81] Schrödinger, LLC. The PyMOL Molecular Graphics System, Version 1.3; 2010.

[82] Kuei C, Yu J, Zhu J, Wu J, Zhang L, Shih A, et al Study of GPR81, the lactate receptor, from distant species identifies residues and motifs critical for GPR81 functions. Mol Pharmacol. 2011 11;80(5):848–858. 10.1124/mol.111.074500 21862690

[83] Li Y, Zhu F, Vaidehi N, Goddard WA, Sheinerman F, Reiling S, et al Prediction of the 3D structure and dynamics of human DP G-protein coupled receptor bound to an agonist and an antagonist. J Am Chem Soc. 2007 9;129(35):10720–10731. 10.1021/ja070865d 17691773 PMC2535578

[84] Wolberger C, Vershon AK, Liu B, Johnson AD, Pabo CO. Crystal structure of a MAT alpha 2 homeodomain-operator complex suggests a general model for homeodomain-DNA interactions. Cell. 1991 11;67(3):517–528. 10.1016/0092-8674(91)90526-5 1682054

[85] Madabushi S, Gross AK, Philippi A, Meng EC, Wensel TG, Lichtarge O. Evolutionary trace of G protein-coupled receptors reveals clusters of residues that determine global and class-specific functions. J Biol Chem. 2004 2;279(9):8126–8132. 14660595 10.1074/jbc.M312671200

[86] Krishnan A, Almen MS, Fredriksson R, Schioth HB. The origin of GPCRs: identification of mammalian like Rhodopsin, Adhesion, Glutamate and Frizzled GPCRs in fungi. PLoS ONE. 2012;7(1):e29817 10.1371/journal.pone.0029817 22238661 PMC3251606

[87] Scott CE, Abrol R, Ahn KH, Kendall DA, Goddard WA. Molecular basis for dramatic changes in cannabinoid CB1 G protein-coupled receptor activation upon single and double point mutations. Protein Sci. 2013 1;22(1):101–113. 10.1002/pro.2192 23184890 PMC3575865

[88] Ahn KH, Scott CE, Abrol R, Goddard WA, Kendall DA. Computationally-predicted CB1 cannabinoid receptor mutants show distinct patterns of salt-bridges that correlate with their level of constitutive activity reflected in G protein coupling levels, thermal stability, and ligand binding. Proteins. 2013 8;81(8):1304–1317. 10.1002/prot.24264 23408552 PMC4872635

[89] Feldman BJ, Rosenthal SM, Vargas GA, Fenwick RG, Huang EA, Matsuda-Abedini M, et al Nephrogenic syndrome of inappropriate antidiuresis. N Engl J Med. 2005 5;352(18):1884–1890. 10.1056/NEJMoa042743 15872203 PMC5340184

[90] Farzan M, Choe H, Martin KA, Sun Y, Sidelko M, Mackay CR, et al HIV-1 entry and macrophage inflammatory protein-1beta-mediated signaling are independent functions of the chemokine receptor CCR5. J Biol Chem. 1997 3;272(11):6854–6857. 10.1074/jbc.272.11.6854 9054370

[91] Beau I, Touraine P, Meduri G, Gougeon A, Desroches A, Matuchansky C, et al A novel phenotype related to partial loss of function mutations of the follicle stimulating hormone receptor. J Clin Invest. 1998 10;102(7):1352–1359. 9769327 10.1172/JCI3795 PMC508982

[92] Schipani E, Langman CB, Parfitt AM, Jensen GS, Kikuchi S, Kooh SW, et al Constitutively activated receptors for parathyroid hormone and parathyroid hormone-related peptide in Jansen’s metaphyseal chondrodysplasia. N Engl J Med. 1996 9;335(10):708–714. 10.1056/NEJM199609053351004 8703170

[93] Bastepe M, Raas-Rothschild A, Silver J, Weissman I, Wientroub S, Juppner H, et al A form of Jansen’s metaphyseal chondrodysplasia with limited metabolic and skeletal abnormalities is caused by a novel activating parathyroid hormone (PTH)/PTH-related peptide receptor mutation. J Clin Endocrinol Metab. 2004 7;89(7):3595–3600. 10.1210/jc.2004-0036 15240651

[94] Watanabe T, Bai M, Lane CR, Matsumoto S, Minamitani K, Minagawa M, et al Familial hypoparathyroidism: identification of a novel gain of function mutation in transmembrane domain 5 of the calcium-sensing receptor. J Clin Endocrinol Metab. 1998 7;83(7):2497–2502. 10.1210/jc.83.7.2497 9661634

[95] Baron J, Winer KK, Yanovski JA, Cunningham AW, Laue L, Zimmerman D, et al Mutations in the Ca(2+)-sensing receptor gene cause autosomal dominant and sporadic hypoparathyroidism. Hum Mol Genet. 1996 5;5(5):601–606. 8733126 10.1093/hmg/5.5.601

[96] De Luca F, Ray K, Mancilla EE, Fan GF, Winer KK, Gore P, et al Sporadic hypoparathyroidism caused by de Novo gain-of-function mutations of the Ca(2+)-sensing receptor. J Clin Endocrinol Metab. 1997 8;82(8):2710–2715. 10.1210/jc.82.8.2710 9253358

[97] Hendy GN, Minutti C, Canaff L, Pidasheva S, Yang B, Nouhi Z, et al Recurrent familial hypocalcemia due to germline mosaicism for an activating mutation of the calcium-sensing receptor gene. J Clin Endocrinol Metab. 2003 8;88(8):3674–3681. 10.1210/jc.2003-030409 12915654

[98] Sjoblom T, Jones S, Wood LD, Parsons DW, Lin J, Barber TD, et al The consensus coding sequences of human breast and colorectal cancers. Science. 2006 10;314(5797):268–274. 10.1126/science.1133427 16959974

[99] Schipani E, Kruse K, Juppner H. A constitutively active mutant PTH-PTHrP receptor in Jansen-type metaphyseal chondrodysplasia. Science. 1995 4;268(5207):98–100. 10.1126/science.7701349 7701349

[100] Coster M, Wittkopf D, Kreuchwig A, Kleinau G, Thor D, Krause G, et al Using ortholog sequence data to predict the functional relevance of mutations in G-protein-coupled receptors. FASEB J. 2012 8;26(8):3273–3281. 10.1096/fj.12-203737 22611087

[101] Pryszcz LP, Huerta-Cepas J, Gabaldon T. MetaPhOrs: orthology and paralogy predictions from multiple phylogenetic evidence using a consistency-based confidence score. Nucleic Acids Res. 2011 3;39(5):e32 10.1093/nar/gkq953 21149260 PMC3061081

[102] Kenakin T, Miller LJ. Seven transmembrane receptors as shapeshifting proteins: the impact of allosteric modulation and functional selectivity on new drug discovery. Pharmacol Rev. 2010 6;62(2):265–304. 10.1124/pr.108.000992 20392808 PMC2879912

[103] Vaidehi N, Kenakin T. The role of conformational ensembles of seven transmembrane receptors in functional selectivity. Curr Opin Pharmacol. 2010 12;10(6):775–781. 10.1016/j.coph.2010.09.004 20933468

[104] Abrol R, Kim SK, Bray JK, Trzaskowski B, Goddard WA. Conformational ensemble view of G protein-coupled receptors and the effect of mutations and ligand binding. Meth Enzymol. 2013;520:31–48. 10.1016/B978-0-12-391861-1.00002-2 23332694

[105] Manglik A, Kobilka B. The role of protein dynamics in GPCR function: insights from the *β*2AR and rhodopsin. Curr Opin Cell Biol. 2014 4;27:136–143. 10.1016/j.ceb.2014.01.008 24534489 PMC3986065

[106] Abrol R, Trzaskowski B, Goddard WA, Nesterov A, Olave I, Irons C. Ligand- and mutation-induced conformational selection in the CCR5 chemokine G protein-coupled receptor. Proc Natl Acad Sci USA. 2014 9;111(36):13040–13045. 10.1073/pnas.1413216111 25157173 PMC4246978

[107] Kim SK, Riley L, Abrol R, Jacobson KA, Goddard WA. Predicted structures of agonist and antagonist bound complexes of adenosine A3 receptor. Proteins. 2011 6;79(6):1878–1897. 10.1002/prot.23012 21488099 PMC3092833

