# Supplementary material for: Structure-Based Sequence Alignment of the Transmembrane Domains of All Human GPCRs: Phylogenetic, Structural and Functional Implications
Source: PLoS Comput Biol. 2016 Mar 30;12(3):e1004805. doi: 10.1371/journal.pcbi.1004805 (PMC4814114; doi:10.1371/journal.pcbi.1004805)
Supplement: S1 Table — When multiple structures are available, then the one with the highest resolution or the one with least deformed TM helices is used. (PDF) [file pcbi.1004805.s001.pdf]

Class	Ligand	Protein Name	Short	Gene	PDB Id Inactive	PDB Id Active	Uniprot	Ref.
Aα	opsin	bovine rhodopsin	RHO	RHO	1GZM.A	3PQR.A	P08100	[38, 39]
Aα	aminergic	turkey β_1 adrenergic receptor	β_1AR	ADRB1	2VT4.B		P08588	[46]
Aα	aminergic	human β_2 adrenergic receptor	β_2AR	ADRB2	2RH1.A	3SN6.R	P07550	[6, 40]
Aα	aminergic	human dopamine D3 receptor	D3	DRD3	3PBL.A		P35462	[47]
Aα	aminergic	human histamine H1 receptor	H1	HRH1	3RZE.A		P35367	[48]
Aα	aminergic	human muscarinic acetylcholine receptor M2	M2	CHRM2	3UON.A	4MQS.A	P08172	[41, 42]
Aα	aminergic	rat muscarinic acetylcholine receptor M3	M3	CHRM3	4DAJ.A		P20309	[49]
Aα	aminergic	human serotonin receptor 1B	5HT1B	HTR1B	4IAR.A		P28222	[50]
Aα	aminergic	human serotonin receptor 2B	5HT2B	HTR2B	4NC3.A		P41595	[51]
Aα	nucleoside	human adenosine receptor A2a	A2A	ADORA2A	3EML.A	3QAK.A	P29274	[43, 44]
Aα	lipid	human sphingosine 1-phosphate receptor 1	S1P1	S1PR1	3V2Y.A		P21453	[52]
Aβ	peptide	rat neurotensin receptor type 1	NTS1	NTSR1		4GRV.A	P30989	[45]
Aγ	peptide	human C-X-C chemokine receptor 4	CXCR4	CXCR4	3ODU.A		P61073	[53]
Aγ	peptide	human C-C chemokine receptor 5	CCR5	CCR5	4MBS.A		P51681	[54]
Aγ	peptide	human κ-opioid receptor	κOR	OPRK1	4DJH.A		P41145	[55]
Aγ	peptide	mouse μ-opioid receptor	μOR	OPRM1	4DKL.A		P35372	[56]
Aγ	peptide	human nociceptin/orphanin FQ opioid receptor	NOP	OPRL1	4EA3.A		P41146	[57]
Aγ	peptide	mouse δ-opioid receptor	δOR	OPRD1	4EJ4.A		P41143	[58]
Aδ	peptide	human proteinase-activated receptor 1	PAR1	F2R	3VW7.A		P25116	[59]
Aδ	nucleoside	human P2Y purinoreceptor 12	P2Y12	P2RY12	4NTJ.A		Q9H244	[60]
B	peptide	human corticotropin-releasing factor receptor 1	CRF1	CRHR1	4K5Y.B		P34998	[61]
B	peptide	human glucagon receptor	GLR	GCGR	4L6R.A		P47871	[62]
C	peptide	human metabotropic glutamate receptor 1	MGLU1	GRM1	4OR2.A		Q13255	[63]
C	peptide	human metabotropic glutamate receptor 5	MGLU5	GRM5	4OO9.A		P41594	[64]
Frizzled	peptide	human smoothened homolog	SMO	SMO	4N4W.A		Q99835	[65, 66]
