# Supplementary material for: Structure-Based Sequence Alignment of the Transmembrane Domains of All Human GPCRs: Phylogenetic, Structural and Functional Implications
Source: PLoS Comput Biol. 2016 Mar 30;12(3):e1004805. doi: 10.1371/journal.pcbi.1004805 (PMC4814114; doi:10.1371/journal.pcbi.1004805)
Supplement: S4 Table — For orthologs of several proteins we computed average amino acids conservation over TM, and over CHICO/NACHO residues. The data shows that CHICO and NACHO positions are more conserved than other TM residues in all GPCR classes. Residues present on both lists are even more conserved. Two measures of conservation provided by Jalview are used: Consensus is the percentage of orthologs sharing the human amino acid; and Conservation is a qualitative measure counting the number of conserved chemical properties. For P2Y12, we used a curated list of 77 orthologs from [100]. For other proteins, we collected predicted orthologs from the MetaPhOrs database (release 201405 [101]), aligned them with Clustal Omega, and then removed sequences with gaps in the TM regions. (PDF) [file pcbi.1004805.s004.pdf]

	Aδ P2Y12	Aδ P2Y12	Aα Beta2AR	Aβ NTS1	Aγ CCR5	B CRF1	C MGLU1	F SMO
Number of orthologs	77 (ref. [100])	42	37	21	24	72	55	48
				Consensus %				
TM	89.8	89	91.6	86.4	83.8	95.6	95	86.7
CHICO	95.1	93.6	97.3	91.1	92.1	98	97.6	88.3
NACHO	92.7	91.3	98.9	94	90.6	98.6	99.1	93.6
CHICO and NACHO	97.8	95.7	99.5	89.5	97.5	99.4	98.9	93.8
			Average conservation (Jalview scale 0-11)					
TM	8.9	9.1	9.5	8.4	7.8	9.8	9.8	8.8
CHICO	9.8	9.9	10.5	9.3	9.5	10.3	9.9	9.2
NACHO	9.7	9.8	10.8	9.1	9.2	10.4	10.4	9.8
CHICO and NACHO	10.4	10.4	10.8	9.8	10.6	10.6	10.4	10
