# Supplementary material for: Structure-Based Sequence Alignment of the Transmembrane Domains of All Human GPCRs: Phylogenetic, Structural and Functional Implications
Source: PLoS Comput Biol. 2016 Mar 30;12(3):e1004805. doi: 10.1371/journal.pcbi.1004805 (PMC4814114; doi:10.1371/journal.pcbi.1004805)
Supplement: S1 Fig — The conserved residues in 24 different structures (including active) have very similar positions, which shows that the class A GPCR fold is highly conserved. The full TM bundle is shown in Fig 1. (PDF) [file pcbi.1004805.s005.pdf]

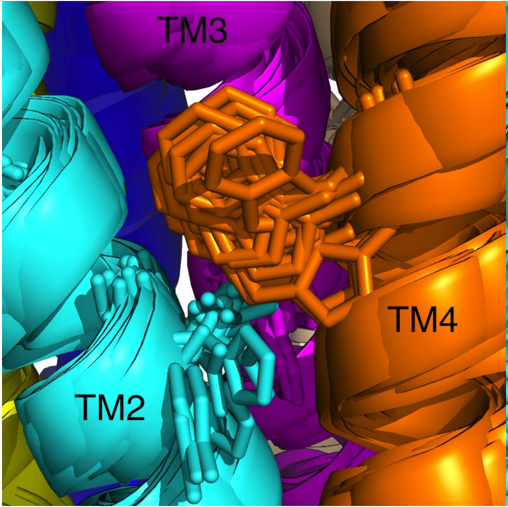

(a) W4.50 ↔ S/N/T2.45 ↔ S/N/T3.42

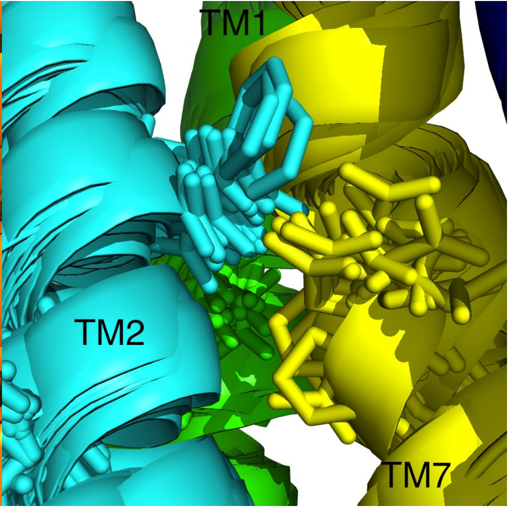

(b) N1.50 ↔ D2.50 ↔ N7.49 (water-mediated)
