# Supplementary material for: Structure-Based Sequence Alignment of the Transmembrane Domains of All Human GPCRs: Phylogenetic, Structural and Functional Implications
Source: PLoS Comput Biol. 2016 Mar 30;12(3):e1004805. doi: 10.1371/journal.pcbi.1004805 (PMC4814114; doi:10.1371/journal.pcbi.1004805)
Supplement: S2 Fig — Two residues are similar if their BLOSUM62 entry is positive. (PDF) [file pcbi.1004805.s006.pdf]

	RHO	RHOact	Beta1AR	Beta2AR	Beta2ARact	D3	H1	M2	M2act	M3	5HT1B	5HT2B	A2A	A2Aact	S1P1	NTS1act	CXCR4	CCR5	KappaOR	MuOR	NOP	DeltaOR	PAR1	P2Y12	CRF1	GLR	MGLU1	MGLU5	SMO
RHO	100.0	100.0	42.9	44.5	44.5	46.7	44.2	42.9	43.8	42.0	46.0	45.0	46.0	45.3	40.0	44.9	40.4	45.8	46.8	47.8	40.9	44.9	38.4	35.0	31.6	32.1	27.3	25.8	25.1
RHOact	100.0	100.0	43.3	44.1	43.7	47.2	44.2	42.9	43.6	42.0	46.0	45.1	46.0	45.1	40.3	44.4	40.2	45.3	45.7	47.3	40.6	44.4	38.9	34.3	30.9	31.0	27.4	26.0	24.9
Beta1AR	42.9	43.3	100.0	82.8	82.8	61.7	62.2	55.6	55.0	56.7	61.8	58.0	54.4	53.8	48.7	48.5	50.5	48.2	52.7	49.7	52.3	52.8	45.7	45.1	31.0	34.4	26.1	24.7	29.3
Beta2AR	44.5	44.1	82.8	100.0	99.5	62.4	62.4	56.1	55.9	57.9	63.7	58.9	51.0	50.5	49.2	46.0	49.2	47.5	50.5	49.3	48.8	50.5	44.3	43.1	28.6	31.8	22.7	24.2	24.9
Beta2ARact	44.5	43.7	82.8	99.5	100.0	62.9	62.4	56.3	55.7	57.2	64.5	58.6	51.0	50.5	49.2	45.7	48.7	47.2	49.3	49.0	48.3	50.8	43.8	43.2	29.0	32.7	22.9	24.3	24.5
D3	46.7	47.2	61.7	62.4	62.9	100.0	59.0	52.8	53.8	55.6	63.6	61.9	51.5	51.3	47.9	49.5	47.4	44.7	50.0	50.8	48.0	50.3	42.9	41.1	29.5	29.6	26.1	25.3	23.9
H1	44.2	44.2	62.2	62.4	62.4	59.0	100.0	57.0	57.4	57.4	59.9	59.0	49.2	49.7	45.2	46.8	46.4	45.6	53.6	52.6	52.0	53.1	49.0	42.3	30.6	36.0	27.3	27.0	30.2
M2	42.9	42.9	55.6	56.1	56.3	52.8	57.0	100.0	100.0	85.4	56.3	53.8	50.8	50.8	47.9	48.5	44.1	45.7	51.0	49.0	48.0	51.0	43.4	39.7	28.1	29.5	29.2	29.4	26.7
M2act	43.8	43.6	55.0	55.9	55.7	53.8	57.4	100.0	100.0	85.6	55.8	54.0	51.3	50.7	46.9	48.5	44.7	45.0	50.5	48.5	47.5	50.8	44.1	39.5	28.9	29.4	29.4	29.6	26.8
M3	42.0	42.0	56.7	57.9	57.2	55.6	57.4	85.4	85.6	100.0	53.6	53.9	50.8	50.5	45.7	48.9	44.5	45.6	50.0	47.2	45.9	48.7	46.4	39.6	30.2	27.5	29.9	30.1	27.9
5HT1B	46.0	46.0	61.8	63.7	64.5	63.6	59.9	56.3	55.8	53.6	100.0	59.9	50.3	49.5	49.7	49.0	46.1	40.0	53.2	50.3	47.2	51.0	40.4	39.2	29.1	33.7	23.3	25.3	25.3
5HT2B	45.0	45.1	58.0	58.9	58.6	61.9	59.0	53.8	54.0	53.9	59.9	100.0	49.5	49.5	48.2	45.3	44.8	44.4	49.5	48.8	48.6	49.5	40.8	40.6	30.9	31.1	26.7	28.1	24.1
A2A	46.0	46.0	54.4	51.0	51.0	51.5	49.2	50.8	51.3	50.8	50.3	49.5	100.0	100.0	47.4	46.6	44.7	44.7	49.7	47.5	45.8	48.5	40.3	40.6	31.9	35.1	26.6	24.2	26.5
A2Aact	45.3	45.1	53.8	50.5	50.5	51.3	49.7	50.8	50.7	50.5	49.5	49.5	100.0	100.0	46.9	45.9	44.3	45.0	49.3	47.3	45.1	48.2	40.2	40.0	32.1	34.6	26.7	24.2	26.6
S1P1	40.0	40.3	48.7	49.2	49.2	47.9	45.2	47.9	46.9	45.7	49.7	48.2	47.4	46.9	100.0	41.9	42.3	43.3	46.4	44.8	43.3	44.4	39.2	39.5	29.1	29.3	28.2	27.4	25.4
NTS1act	44.9	44.4	48.5	46.0	45.7	49.5	46.8	48.5	48.5	48.9	49.0	45.3	46.6	45.9	41.9	100.0	47.5	40.7	49.3	48.5	49.3	49.0	43.3	41.5	24.2	26.4	21.8	18.8	21.7
CXCR4	40.4	40.2	50.5	49.2	48.7	47.4	46.4	44.1	44.7	44.5	46.1	44.8	44.7	44.3	42.3	47.5	100.0	62.3	55.9	53.4	53.9	54.5	48.5	50.0	31.7	32.6	21.7	22.6	25.0
CCR5	45.8	45.3	48.2	47.5	47.2	44.7	45.6	45.7	45.0	45.6	40.0	44.4	44.7	45.0	43.3	40.7	62.3	100.0	57.8	55.6	52.2	55.1	52.2	51.2	33.7	34.2	24.9	22.9	28.4
KappaOR	46.8	45.7	52.7	50.5	49.3	50.0	53.6	51.0	50.5	50.0	53.2	49.5	49.7	49.3	46.4	49.3	55.9	57.8	100.0	86.5	76.6	84.8	52.2	49.3	35.5	33.2	24.7	23.6	29.2
MuOR	47.8	47.3	49.7	49.3	49.0	50.8	52.6	49.0	48.5	47.2	50.3	48.8	47.5	47.3	44.8	48.5	53.4	55.6	86.5	100.0	74.8	83.1	49.0	47.5	33.5	32.8	22.0	22.9	25.9
NOP	40.9	40.6	52.3	48.8	48.3	48.0	52.0	48.0	47.5	45.9	47.2	48.6	45.8	45.1	43.3	49.3	53.9	52.2	76.6	74.8	100.0	73.9	50.7	46.1	31.9	32.2	23.7	22.9	25.4
DeltaOR	44.9	44.4	52.8	50.5	50.8	50.3	53.1	51.0	50.8	48.7	51.0	49.5	48.5	48.2	44.4	49.0	54.5	55.1	84.8	83.1	73.9	100.0	51.5	44.6	32.8	33.0	24.2	24.4	28.7
PAR1	38.4	38.9	45.7	44.3	43.8	42.9	49.0	43.4	44.1	46.4	40.4	40.8	40.3	40.2	39.2	43.3	48.5	52.2	52.2	49.0	50.7	51.5	100.0	48.5	32.8	32.7	23.7	22.3	27.5
P2Y12	35.0	34.3	45.1	43.1	43.2	41.1	42.3	39.7	39.5	39.6	39.2	40.6	40.6	40.0	39.5	41.5	50.0	51.2	49.3	47.5	46.1	44.6	48.5	100.0	35.0	33.0	29.1	27.1	27.2
CRF1	31.6	30.9	31.0	28.6	29.0	29.5	30.6	28.1	28.9	30.2	29.1	30.9	31.9	32.1	29.1	24.2	31.7	33.7	35.5	33.5	31.9	32.8	32.8	35.0	100.0	58.0	28.4	29.8	33.0
GLR	32.1	31.0	34.4	31.8	32.7	29.6	36.0	29.5	29.4	27.5	33.7	31.1	35.1	34.6	29.3	26.4	32.6	34.2	33.2	32.8	32.2	33.0	32.7	33.0	58.0	100.0	25.6	26.9	30.6
MGLU1	27.3	27.4	26.1	22.7	22.9	26.1	27.3	29.2	29.4	29.9	23.3	26.7	26.6	26.7	28.2	21.8	21.7	24.9	24.7	22.0	23.7	24.2	23.7	29.1	28.4	25.6	100.0	85.9	31.0
MGLU5	25.8	26.0	24.7	24.2	24.3	25.3	27.0	29.4	29.6	30.1	25.3	28.1	24.2	24.2	27.4	18.8	22.6	22.9	23.6	22.9	22.9	24.4	22.3	27.1	29.8	26.9	85.9	100.0	28.9
SMO	25.1	24.9	29.3	24.9	24.5	23.9	30.2	26.7	26.8	27.9	25.3	24.1	26.5	26.6	25.4	21.7	25.0	28.4	29.2	25.9	25.4	28.7	27.5	27.2	33.0	30.6	31.0	28.9	100.0
