# Supplementary material for: Structure-Based Sequence Alignment of the Transmembrane Domains of All Human GPCRs: Phylogenetic, Structural and Functional Implications
Source: PLoS Comput Biol. 2016 Mar 30;12(3):e1004805. doi: 10.1371/journal.pcbi.1004805 (PMC4814114; doi:10.1371/journal.pcbi.1004805)
Supplement: S3 Fig — For a given pair of structures, there may exist a different sequence alignment, which results in a lower RMSD than the listed one. (PDF) [file pcbi.1004805.s007.pdf]

	RHO	RHOact	Beta1AR	Beta2AR	Beta2ARact	D3	H1	M2	M2act	M3	5HT1B	5HT2B	A2A	A2Aact	S1P1	NTS1act	CXCR4	CCR5	KappaOR	MuOR	NOP	DeltaOR	PAR1	P2Y12	CRF1	GLR	MGLU1	MGLU5	SMO
RHO	0.00	2.22	2.12	2.07	3.21	1.68	1.96	2.25	2.66	2.40	2.15	2.00	2.36	2.72	2.26	2.91	2.16	2.08	2.50	1.85	1.86	1.99	2.62	2.91	3.21	2.88	3.14	3.25	3.03
RHOact	2.22	0.00	2.49	2.35	2.05	2.53	2.28	2.66	1.84	2.31	1.99	2.09	2.69	2.40	2.74	2.41	2.80	2.52	3.02	2.68	2.84	2.50	2.47	2.90	3.45	3.01	3.09	3.23	3.15
Beta1AR	2.12	2.49	0.00	0.60	2.54	1.38	1.53	1.34	2.09	1.53	1.51	1.91	1.89	2.58	1.77	2.70	2.75	2.24	2.32	2.32	2.20	2.21	2.96	3.14	3.14	2.85	2.95	3.11	3.09
Beta2AR	2.07	2.35	0.60	0.00	2.45	1.36	1.38	1.40	2.01	1.42	1.35	1.83	1.86	2.48	1.86	2.52	2.64	2.26	2.16	2.26	2.10	2.09	2.86	3.08	3.06	2.70	2.89	3.06	3.09
Beta2ARact	3.21	2.05	2.54	2.45	0.00	3.11	2.90	2.63	1.38	1.99	1.66	2.49	3.09	2.78	3.46	2.18	3.48	3.15	3.29	3.43	3.34	2.65	3.04	2.59	3.81	3.38	3.14	3.36	3.57
D3	1.68	2.53	1.38	1.36	3.11	0.00	1.36	1.66	2.36	1.80	1.44	1.71	1.58	2.12	1.61	2.36	2.01	1.80	1.84	1.64	1.58	1.70	2.75	2.79	2.63	2.54	2.79	2.91	2.44
H1	1.96	2.28	1.53	1.38	2.90	1.36	0.00	1.48	2.19	1.35	1.44	1.75	1.94	2.35	1.87	2.43	2.12	1.79	1.83	1.79	1.71	1.69	2.65	2.76	2.81	2.67	2.89	2.98	2.56
M2	2.25	2.66	1.34	1.40	2.63	1.66	1.48	0.00	2.23	1.10	1.96	2.12	2.06	2.72	1.95	2.96	2.52	2.13	2.19	2.12	2.06	2.08	3.26	3.16	3.16	3.06	3.04	3.19	2.97
M2act	2.66	1.84	2.09	2.01	1.38	2.36	2.19	2.23	0.00	1.72	1.31	1.89	2.44	2.14	2.68	2.02	3.12	2.88	2.82	2.89	2.72	2.55	2.83	2.87	3.15	3.19	2.89	3.09	3.40
M3	2.40	2.31	1.53	1.42	1.99	1.80	1.35	1.10	1.72	0.00	1.77	1.99	2.03	2.48	1.97	2.62	2.70	2.29	2.29	2.40	2.23	2.22	3.13	3.14	3.12	3.01	2.86	3.04	3.05
5HT1B	2.15	1.99	1.51	1.35	1.66	1.44	1.44	1.96	1.31	1.77	0.00	1.64	1.92	2.01	2.15	1.99	2.59	2.37	2.33	2.35	2.21	2.24	2.68	2.97	2.83	2.76	2.75	2.90	3.03
5HT2B	2.00	2.09	1.91	1.83	2.49	1.71	1.75	2.12	1.89	1.99	1.64	0.00	2.07	2.07	2.25	2.40	2.55	2.13	2.26	2.29	2.38	2.31	2.84	3.14	3.02	2.85	2.85	2.99	2.86
A2A	2.36	2.69	1.89	1.86	3.09	1.58	1.94	2.06	2.44	2.03	1.92	2.07	0.00	1.63	1.70	2.51	2.65	2.22	2.53	2.33	2.38	2.27	3.16	3.18	3.02	3.03	2.96	3.06	3.00
A2Aact	2.72	2.40	2.58	2.48	2.78	2.12	2.35	2.72	2.14	2.48	2.01	2.07	1.63	0.00	2.34	2.08	2.98	2.79	3.07	2.83	2.85	2.75	3.06	3.10	2.81	3.07	3.00	3.15	3.11
S1P1	2.26	2.74	1.77	1.86	3.46	1.61	1.87	1.95	2.68	1.97	2.15	2.25	1.70	2.34	0.00	3.02	2.60	2.12	2.74	2.24	2.15	2.28	3.07	3.19	2.76	2.90	2.74	2.83	2.81
NTS1act	2.91	2.41	2.70	2.52	2.18	2.36	2.43	2.96	2.02	2.62	1.99	2.40	2.51	2.08	3.02	0.00	2.89	3.06	2.63	2.82	2.67	2.52	2.43	2.83	2.99	2.90	3.00	3.19	3.31
CXCR4	2.16	2.80	2.75	2.64	3.48	2.01	2.12	2.52	3.12	2.70	2.59	2.55	2.65	2.98	2.60	2.89	0.00	1.79	2.32	1.78	1.97	1.87	2.89	2.81	3.13	2.93	3.14	3.25	2.52
CCR5	2.08	2.52	2.24	2.26	3.15	1.80	1.79	2.13	2.88	2.29	2.37	2.13	2.22	2.79	2.12	3.06	1.79	0.00	2.41	1.74	1.83	1.79	2.75	3.16	3.24	3.04	3.12	3.20	2.80
KappaOR	2.50	3.02	2.32	2.16	3.29	1.84	1.83	2.19	2.82	2.29	2.33	2.26	2.53	3.07	2.74	2.63	2.32	2.41	0.00	1.41	1.59	1.45	2.80	3.46	3.25	2.88	3.16	3.38	3.25
MuOR	1.85	2.68	2.32	2.26	3.43	1.64	1.79	2.12	2.89	2.40	2.35	2.29	2.33	2.83	2.24	2.82	1.78	1.74	1.41	0.00	1.02	0.84	2.77	2.91	2.96	2.64	3.11	3.27	2.66
NOP	1.86	2.84	2.20	2.10	3.34	1.58	1.71	2.06	2.72	2.23	2.21	2.38	2.38	2.85	2.15	2.67	1.97	1.83	1.59	1.02	0.00	1.09	2.59	3.24	2.99	2.73	3.06	3.20	2.82
DeltaOR	1.99	2.50	2.21	2.09	2.65	1.70	1.69	2.08	2.55	2.22	2.24	2.31	2.27	2.75	2.28	2.52	1.87	1.79	1.45	0.84	1.09	0.00	2.52	2.67	3.09	2.75	3.12	3.32	2.70
PAR1	2.62	2.47	2.96	2.86	3.04	2.75	2.65	3.26	2.83	3.13	2.68	2.84	3.16	3.06	3.07	2.43	2.89	2.75	2.80	2.77	2.59	2.52	0.00	2.23	3.59	3.35	3.43	3.66	3.52
P2Y12	2.91	2.90	3.14	3.08	2.59	2.79	2.76	3.16	2.87	3.14	2.97	3.14	3.18	3.10	3.19	2.83	2.81	3.16	3.46	2.91	3.24	2.67	2.23	0.00	3.43	3.24	3.27	3.52	3.31
CRF1	3.21	3.45	3.14	3.06	3.81	2.63	2.81	3.16	3.15	3.12	2.83	3.02	3.02	2.81	2.76	2.99	3.13	3.24	3.25	2.96	2.99	3.09	3.59	3.43	0.00	2.42	3.17	3.17	2.87
GLR	2.88	3.01	2.85	2.70	3.38	2.54	2.67	3.06	3.19	3.01	2.76	2.85	3.03	3.07	2.90	2.90	2.93	3.04	2.88	2.64	2.73	2.75	3.35	3.24	2.42	0.00	2.85	2.94	2.52
MGLU1	3.14	3.09	2.95	2.89	3.14	2.79	2.89	3.04	2.89	2.86	2.75	2.85	2.96	3.00	2.74	3.00	3.14	3.12	3.16	3.11	3.06	3.12	3.43	3.27	3.17	2.85	0.00	0.73	3.10
MGLU5	3.25	3.23	3.11	3.06	3.36	2.91	2.98	3.19	3.09	3.04	2.90	2.99	3.06	3.15	2.83	3.19	3.25	3.20	3.38	3.27	3.20	3.32	3.66	3.52	3.17	2.94	0.73	0.00	3.13
SMO	3.03	3.15	3.09	3.09	3.57	2.44	2.56	2.97	3.40	3.05	3.03	2.86	3.00	3.11	2.81	3.31	2.52	2.80	3.25	2.66	2.82	2.70	3.52	3.31	2.87	2.52	3.10	3.13	0.00
