# Supplementary figures and images for: Structure-Based Sequence Alignment of the Transmembrane Domains of All Human GPCRs: Phylogenetic, Structural and Functional Implications
Source: PLoS Comput Biol. 2016 Mar 30;12(3):e1004805. doi: 10.1371/journal.pcbi.1004805 (PMC4814114; doi:10.1371/journal.pcbi.1004805)

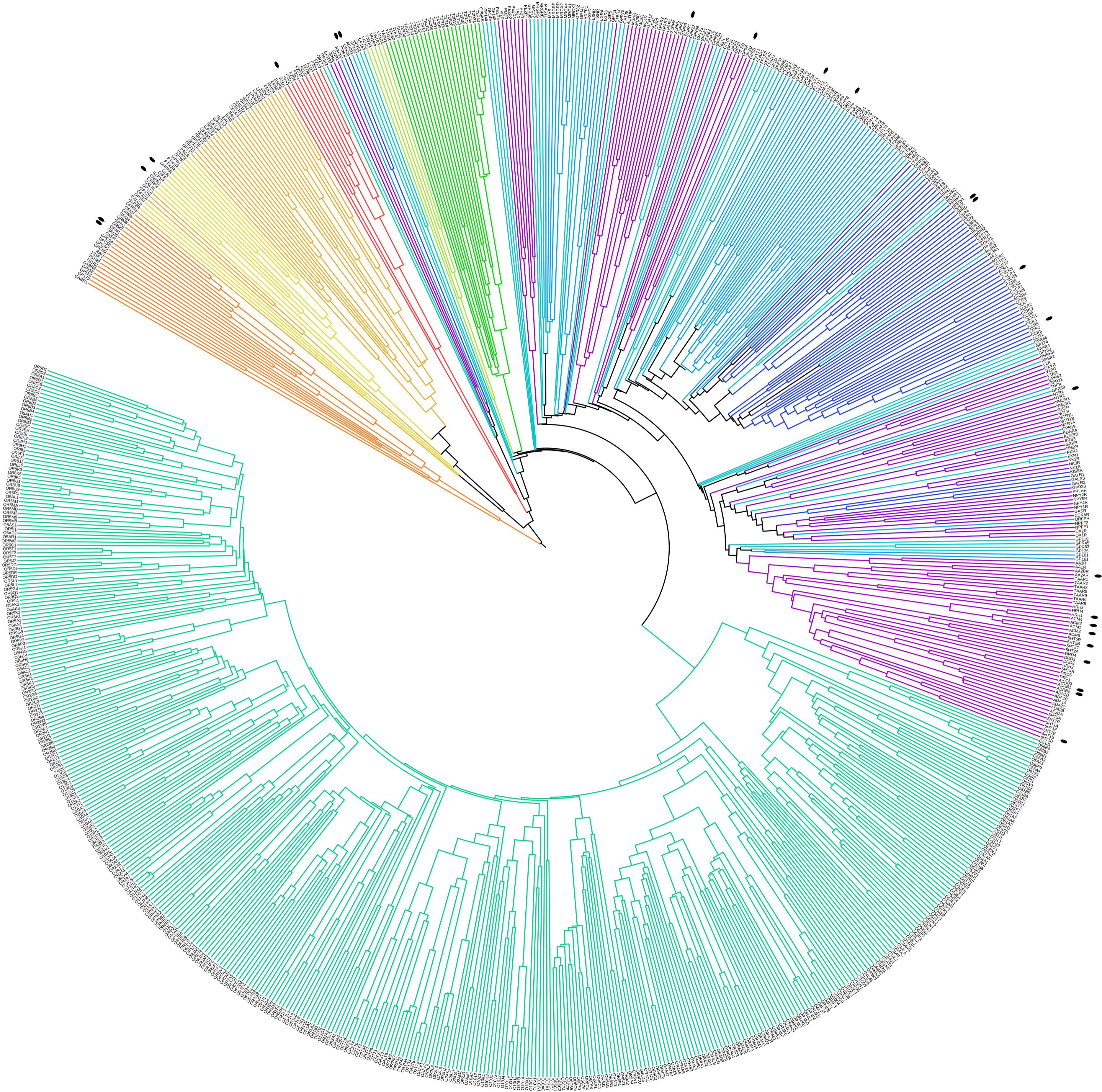

Supplement: S4 Fig — The pdf file is searchable for the UNIPROT accession numbers. Loops were ignored. Color coding denotes the GPCR class. Proteins with known crystal structure are emphasized with a dot. (PDF) [file pcbi.1004805.s008.pdf]

RMSD of helices after best rigid body move

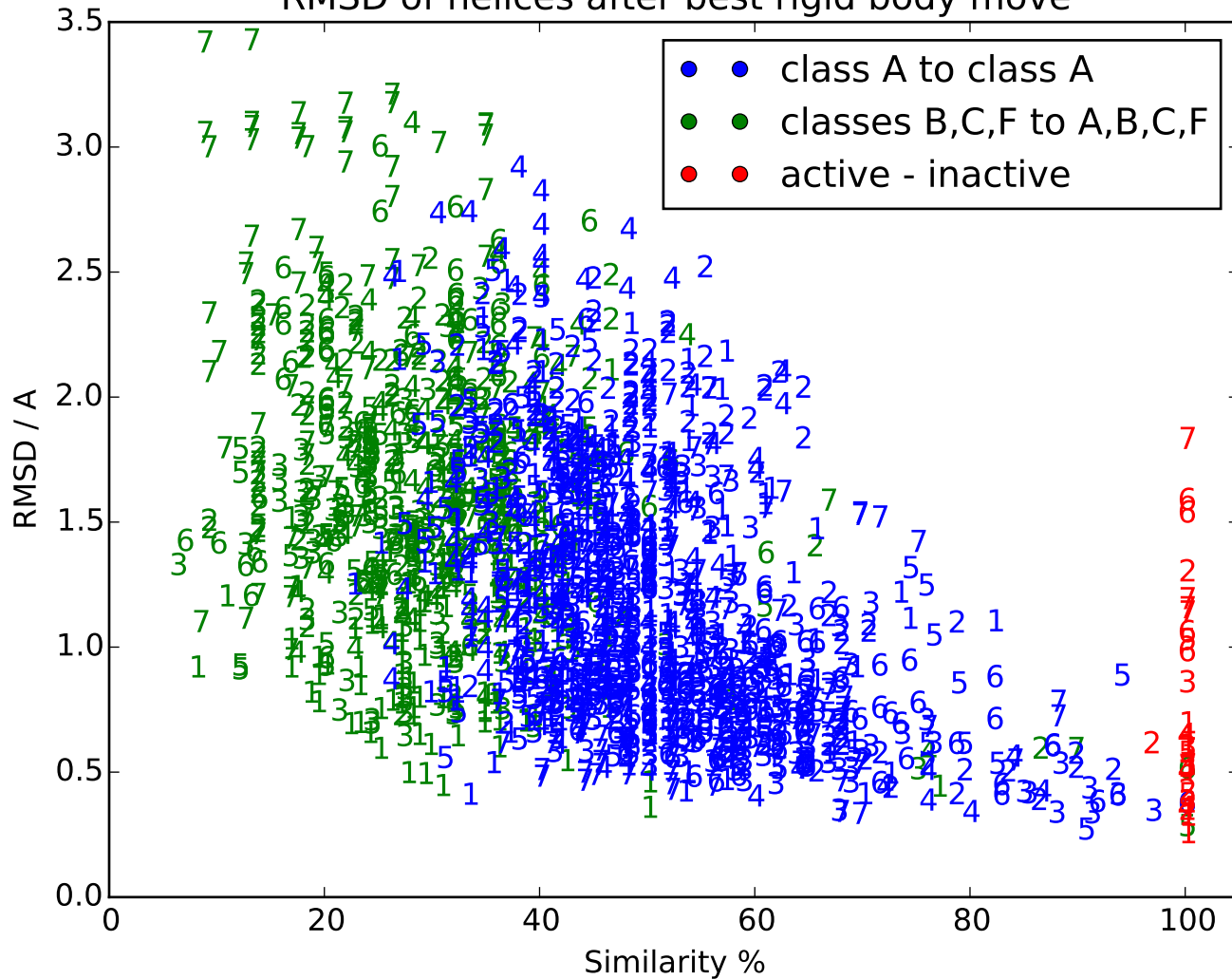

Supplement: S10 Fig — Same caption as Fig 9. (PDF) [file pcbi.1004805.s014.pdf]
