# Supplementary material for: Structure-Based Sequence Alignment of the Transmembrane Domains of All Human GPCRs: Phylogenetic, Structural and Functional Implications
Source: PLoS Comput Biol. 2016 Mar 30;12(3):e1004805. doi: 10.1371/journal.pcbi.1004805 (PMC4814114; doi:10.1371/journal.pcbi.1004805)
Supplement: S5 Fig — This is an extended version of Fig 3, same caption. (PDF) [file pcbi.1004805.s009.pdf]

TM	Vomeronasal similarity to	Offset										
		-5	-4	-3	-2	-1	0	1	2	3	4	5
TM1	A-alpha	0.21	0.20	0.23	0.26	0.29	0.33	0.23	0.22	0.24	0.25	0.23
	A-beta	0.21	0.21	0.25	0.27	0.26	0.34	0.23	0.23	0.27	0.24	0.22
	A-gamma	0.20	0.19	0.25	0.26	0.27	0.38	0.25	0.23	0.25	0.24	0.22
	A-delta	0.20	0.20	0.24	0.26	0.25	0.34	0.24	0.24	0.23	0.23	0.22
	B	0.19	0.26	0.26	0.26	0.21	0.23	0.24	0.26	0.28	0.24	0.24
	C	0.24	0.19	0.26	0.24	0.22	0.35	0.29	0.28	0.27	0.25	0.24
	F	0.16	0.17	0.23	0.19	0.20	0.27	0.26	0.20	0.15	0.19	0.25
TM2	A-alpha	0.21	0.24	0.20	0.27	0.26	0.35	0.23	0.22	0.27	0.23	0.27
	A-beta	0.23	0.23	0.20	0.26	0.28	0.40	0.24	0.22	0.25	0.22	0.27
	A-gamma	0.23	0.21	0.19	0.32	0.20	0.40	0.23	0.26	0.25	0.18	0.29
	A-delta	0.20	0.19	0.21	0.33	0.22	0.38	0.21	0.24	0.23	0.19	0.28
	B	0.20	0.24	0.15	0.25	0.25	0.35	0.20	0.17	0.26	0.25	0.33
	C	0.15	0.19	0.20	0.21	0.30	0.23	0.24	0.23	0.23	0.33	0.24
	F	0.15	0.20	0.17	0.22	0.17	0.30	0.23	0.22	0.28	0.23	0.27
TM3	A-alpha	0.22	0.20	0.21	0.21	0.16	0.36	0.16	0.20	0.21	0.14	0.17
	A-beta	0.18	0.25	0.22	0.21	0.16	0.38	0.18	0.22	0.22	0.13	0.17
	A-gamma	0.15	0.24	0.23	0.18	0.12	0.38	0.19	0.23	0.23	0.15	0.20
	A-delta	0.17	0.21	0.21	0.17	0.12	0.36	0.18	0.23	0.25	0.15	0.20
	B	0.12	0.21	0.19	0.17	0.21	0.26	0.15	0.25	0.18	0.20	0.24
	C	0.17	0.24	0.13	0.25	0.22	0.23	0.14	0.21	0.19	0.19	0.23
	F	0.15	0.20	0.21	0.20	0.25	0.28	0.23	0.20	0.22	0.23	0.19
TM4	A-alpha	0.23	0.23	0.23	0.25	0.25	0.33	0.22	0.23	0.21	0.27	0.19
	A-beta	0.23	0.23	0.22	0.24	0.23	0.31	0.23	0.25	0.22	0.28	0.19
	A-gamma	0.22	0.24	0.26	0.25	0.22	0.34	0.23	0.24	0.25	0.24	0.20
	A-delta	0.21	0.22	0.28	0.26	0.25	0.31	0.24	0.24	0.24	0.24	0.21
	B	0.20	0.19	0.19	0.18	0.20	0.30	0.26	0.18	0.21	0.23	0.19
	C	0.29	0.29	0.27	0.27	0.26	0.30	0.29	0.27	0.27	0.24	0.23
	F	0.15	0.24	0.20	0.19	0.20	0.33	0.26	0.18	0.20	0.29	0.27
TM5	A-alpha	0.20	0.24	0.20	0.22	0.28	0.25	0.21	0.28	0.24	0.21	0.19
	A-beta	0.25	0.23	0.22	0.22	0.27	0.28	0.20	0.25	0.24	0.23	0.20
	A-gamma	0.22	0.23	0.23	0.23	0.26	0.30	0.21	0.24	0.23	0.22	0.20
	A-delta	0.21	0.23	0.23	0.23	0.25	0.30	0.22	0.22	0.23	0.23	0.18
	B	0.25	0.26	0.23	0.22	0.25	0.31	0.29	0.26	0.24	0.27	0.25
	C	0.25	0.25	0.22	0.24	0.24	0.27	0.26	0.24	0.26	0.16	0.15
	F	0.22	0.25	0.24	0.28	0.17	0.24	0.27	0.28	0.25	0.23	0.22
TM6	A-alpha	0.24	0.27	0.28	0.23	0.26	0.39	0.25	0.21	0.26	0.23	0.21
	A-beta	0.24	0.32	0.28	0.24	0.28	0.36	0.28	0.20	0.28	0.23	0.20
	A-gamma	0.25	0.30	0.33	0.24	0.28	0.38	0.27	0.21	0.29	0.24	0.20
	A-delta	0.25	0.28	0.29	0.24	0.26	0.35	0.25	0.22	0.27	0.24	0.22
	B	0.22	0.19	0.24	0.21	0.25	0.39	0.30	0.22	0.36	0.27	0.24
	C	0.18	0.25	0.20	0.28	0.21	0.26	0.28	0.22	0.22	0.26	0.20
	F	0.23	0.29	0.29	0.22	0.21	0.23	0.18	0.23	0.17	0.21	0.19
TM7	A-alpha	0.20	0.21	0.19	0.22	0.24	0.31	0.19	0.21	0.20	0.23	0.16
	A-beta	0.18	0.22	0.20	0.23	0.25	0.32	0.20	0.25	0.26	0.24	0.15
	A-gamma	0.18	0.23	0.21	0.24	0.22	0.33	0.19	0.23	0.22	0.24	0.14
	A-delta	0.18	0.27	0.21	0.21	0.25	0.30	0.20	0.24	0.25	0.27	0.16
	B	0.22	0.23	0.19	0.20	0.24	0.24	0.24	0.19	0.14	0.20	0.14
	C	0.19	0.26	0.23	0.27	0.26	0.26	0.26	0.27	0.28	0.22	0.21
	F	0.26	0.19	0.18	0.16	0.25	0.28	0.27	0.26	0.25	0.19	0.17
