# Supplementary material for: Structure-Based Sequence Alignment of the Transmembrane Domains of All Human GPCRs: Phylogenetic, Structural and Functional Implications
Source: PLoS Comput Biol. 2016 Mar 30;12(3):e1004805. doi: 10.1371/journal.pcbi.1004805 (PMC4814114; doi:10.1371/journal.pcbi.1004805)
Supplement: S8 Fig — Same caption as in Fig 4. (PDF) [file pcbi.1004805.s012.pdf]

Uses GPCRtm substitution matrix

TM	**Taste2 similarity to**	Offset										
		-5	-4	-3	-2	-1	0	1	2	3	4	5
TM1	A-alpha	0.25	0.21	0.28	0.28	0.26	0.34	0.26	0.25	0.27	0.27	0.24
	A-beta	0.27	0.23	0.29	0.29	0.22	0.37	0.25	0.24	0.29	0.29	0.23
	A-gamma	0.26	0.21	0.30	0.25	0.24	0.38	0.23	0.24	0.27	0.28	0.22
	A-delta	0.25	0.21	0.27	0.22	0.24	0.35	0.23	0.24	0.26	0.27	0.24
	B	0.25	0.26	0.28	0.25	0.24	0.32	0.27	0.28	0.26	0.30	0.23
	C	0.27	0.21	0.27	0.23	0.26	0.34	0.26	0.25	0.27	0.27	0.26
	F	0.23	0.16	0.19	0.22	0.26	0.26	0.24	0.24	0.25	0.27	0.25
TM2	A-alpha	0.22	0.21	0.22	0.25	0.23	0.35	0.23	0.24	0.21	0.25	0.28
	A-beta	0.25	0.20	0.25	0.24	0.22	0.36	0.23	0.25	0.21	0.24	0.27
	A-gamma	0.25	0.17	0.22	0.25	0.21	0.34	0.23	0.26	0.20	0.24	0.22
	A-delta	0.25	0.19	0.23	0.25	0.21	0.32	0.23	0.26	0.22	0.25	0.23
	B	0.20	0.20	0.14	0.19	0.23	0.30	0.22	0.21	0.28	0.24	0.26
	C	0.19	0.21	0.18	0.19	0.27	0.25	0.25	0.26	0.28	0.29	0.26
	F	0.19	0.17	0.16	0.19	0.20	0.28	0.24	0.25	0.19	0.26	0.25
TM3	A-alpha	0.22	0.20	0.20	0.22	0.18	0.29	0.20	0.22	0.22	0.20	0.14
	A-beta	0.21	0.22	0.20	0.20	0.18	0.30	0.22	0.20	0.23	0.19	0.15
	A-gamma	0.22	0.20	0.20	0.20	0.19	0.33	0.22	0.19	0.24	0.20	0.17
	A-delta	0.20	0.22	0.20	0.21	0.18	0.32	0.22	0.18	0.22	0.19	0.17
	B	0.17	0.19	0.23	0.19	0.22	0.24	0.23	0.29	0.25	0.19	0.18
	C	0.16	0.24	0.19	0.26	0.21	0.18	0.17	0.26	0.21	0.23	0.19
	F	0.22	0.23	0.23	0.19	0.18	0.27	0.25	0.24	0.20	0.24	0.18
TM4	A-alpha	0.28	0.28	0.26	0.24	0.24	0.29	0.27	0.26	0.20	0.22	0.23
	A-beta	0.29	0.26	0.24	0.23	0.24	0.32	0.27	0.26	0.23	0.26	0.24
	A-gamma	0.23	0.28	0.24	0.26	0.26	0.27	0.25	0.26	0.24	0.25	0.25
	A-delta	0.25	0.26	0.25	0.27	0.28	0.27	0.25	0.25	0.24	0.24	0.24
	B	0.18	0.18	0.24	0.24	0.25	0.26	0.21	0.21	0.17	0.18	0.19
	C	0.28	0.26	0.24	0.27	0.29	0.27	0.27	0.26	0.26	0.24	0.25
	F	0.16	0.28	0.25	0.23	0.19	0.19	0.22	0.25	0.21	0.22	0.26
TM5	A-alpha	0.23	0.27	0.22	0.22	0.26	0.30	0.25	0.23	0.22	0.23	0.23
	A-beta	0.24	0.26	0.22	0.25	0.23	0.33	0.24	0.20	0.23	0.23	0.25
	A-gamma	0.24	0.27	0.22	0.22	0.26	0.35	0.24	0.22	0.24	0.23	0.25
	A-delta	0.22	0.26	0.22	0.23	0.25	0.34	0.23	0.22	0.23	0.24	0.23
	B	0.21	0.29	0.27	0.25	0.24	0.29	0.26	0.23	0.29	0.34	0.23
	C	0.25	0.26	0.24	0.25	0.24	0.21	0.25	0.19	0.20	0.24	0.20
	F	0.19	0.27	0.28	0.26	0.24	0.28	0.20	0.25	0.30	0.26	0.19
TM6	A-alpha	0.24	0.24	0.29	0.24	0.22	0.32	0.24	0.25	0.29	0.31	0.25
	A-beta	0.23	0.29	0.27	0.23	0.20	0.32	0.22	0.23	0.26	0.30	0.25
	A-gamma	0.23	0.26	0.27	0.23	0.22	0.32	0.24	0.24	0.28	0.32	0.26
	A-delta	0.25	0.24	0.27	0.23	0.22	0.32	0.23	0.24	0.27	0.31	0.24
	B	0.29	0.22	0.32	0.30	0.25	0.37	0.30	0.23	0.27	0.32	0.22
	C	0.25	0.26	0.20	0.24	0.24	0.27	0.27	0.23	0.28	0.29	0.27
	F	0.18	0.29	0.29	0.21	0.28	0.31	0.26	0.21	0.22	0.22	0.23
TM7	A-alpha	0.20	0.24	0.21	0.25	0.21	0.27	0.17	0.20	0.17	0.26	0.14
	A-beta	0.18	0.24	0.23	0.26	0.24	0.30	0.19	0.22	0.17	0.23	0.15
	A-gamma	0.18	0.26	0.23	0.28	0.21	0.30	0.17	0.21	0.13	0.26	0.12
	A-delta	0.18	0.27	0.22	0.25	0.20	0.28	0.17	0.20	0.19	0.25	0.14
	B	0.25	0.26	0.22	0.21	0.29	0.23	0.16	0.23	0.24	0.21	0.14
	C	0.24	0.26	0.25	0.25	0.28	0.21	0.26	0.22	0.23	0.22	0.21
	F	0.22	0.20	0.19	0.19	0.26	0.26	0.25	0.22	0.22	0.21	0.20
