# Supplementary material for: Structure-Based Sequence Alignment of the Transmembrane Domains of All Human GPCRs: Phylogenetic, Structural and Functional Implications
Source: PLoS Comput Biol. 2016 Mar 30;12(3):e1004805. doi: 10.1371/journal.pcbi.1004805 (PMC4814114; doi:10.1371/journal.pcbi.1004805)
Supplement: S9 Fig — The width of the line connecting two TMs is proportional to the number of contacts present in all structures from the given class. The list in red font shows the contacts not present in any available structure from other classes. (PDF) [file pcbi.1004805.s013.pdf]

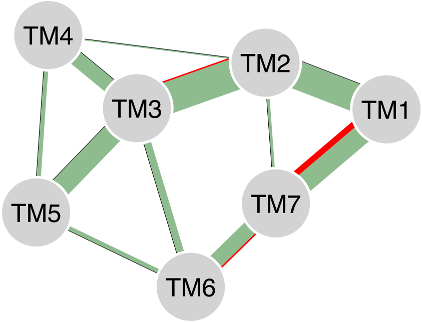

TM2-TM3: 2.63-3.25
TM6-TM7: 6.47-7.38
TM1-TM7: 1.43-7.42, 1.46-7.44, 1.50-7.49, 1.56-7.54

(a) Class B: CRF1, GLR

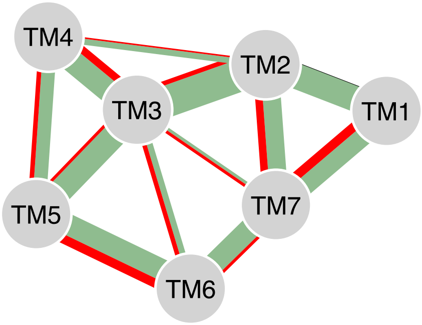

TM2-TM3: 2.48-3.38, 2.52-3.38, 2.59-3.27
TM3-TM4: 3.33-4.53, 3.37-4.50, 3.41-4.46, 3.44-4.49, 3.45-4.42
TM3-TM5: 3.33-5.47, 3.37-5.50
TM3-TM6: 3.40-6.41, 3.43-6.37, 3.46-6.33
TM3-TM7: 3.32-7.38, 3.40-7.45
TM2-TM4: 2.42-4.43
TM4-TM5: 4.49-5.46, 4.53-5.43, 4.53-5.47
TM5-TM6: 5.37-6.56, 5.41-6.56, 5.44-6.48, 5.44-6.51, 5.48-6.48, 5.59-6.42
TM6-TM7: 6.55-7.34, 6.55-7.37
TM2-TM7: 2.46-7.54, 2.50-7.54, 2.54-7.43, 2.54-7.47, 2.60-7.38
TM1-TM7: 1.36-7.36, 1.52-7.58, 1.53-7.58, 1.56-7.58, 1.56-7.61, 1.57-7.58

(b) Class C: MGLU1, MGLU2

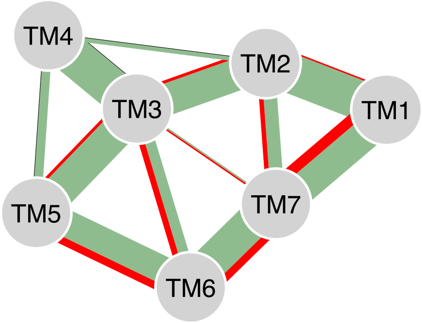

TM2-TM3: 2.50-3.36, 2.57-3.36
TM3-TM5: 3.43-5.50, 3.50-5.62
TM3-TM6: 3.43-6.45, 3.50-6.38, 3.50-6.42, 3.53-6.34
TM3-TM7: 3.46-7.49
TM5-TM6: 5.44-6.59, 5.47-6.51, 5.50-6.48, 5.58-6.45, 5.62-6.42
TM6-TM7: 6.33-7.52, 6.36-7.51, 6.40-7.51, 6.47-7.40, 6.50-7.40
TM2-TM7: 2.40-7.52, 2.50-7.42, 2.57-7.42
TM1-TM2: 1.36-2.60
TM1-TM7: 1.31-7.32, 1.35-7.32, 1.35-7.35, 1.38-7.39, 1.52-7.53, 1.56-7.53, 1.57-7.52

(c) Class F: SMO
