# Supplementary material for: Structure-Based Sequence Alignment of the Transmembrane Domains of All Human GPCRs: Phylogenetic, Structural and Functional Implications
Source: PLoS Comput Biol. 2016 Mar 30;12(3):e1004805. doi: 10.1371/journal.pcbi.1004805 (PMC4814114; doi:10.1371/journal.pcbi.1004805)
Supplement: S1 Text — (DOCX) [file pcbi.1004805.s015.docx]

**“Structure-Based Sequence Alignment of the Transmembrane Domains of All Human GPCRs: Phylogenetic, Structural and Functional Implications”, Cvicek et al.**

**Supporting Text 1**

Here we compare the GRoSS alignment to two other available GPCR alignments:

1. Structure based alignment with gaps developed by Isberg et al. [24], downloaded from GPCRDB [78]
2. HMM-HMM based alignment created by hhalign [77]

**Comparison of GRoSS sequence alignment to HMM-HMM and GPCRDB**

*GRoSS*

Within each GPCR class, the GRoSS alignment is an alignment that preserves the most conserved (BW) residues and has been curated not to contain gaps in the TM regions. First, we used Clustal Omega [37] to align small (~40) groups of proteins together so that no gaps are created in the TM regions. These individual alignments were then aligned together again using Clustal Omega, which uses hidden Markov model for profile-profile alignments. Between classes, the sequences were aligned to maximize the number of conserved contacts.

*HMM-HMM*

We compare our alignment to a general HMM-HMM alignment computed for each target—template pair taken from the available crystal structures. For each crystal structure, we searched for related sequences using hhblitz (<http://toolkit.lmb.uni-muenchen.de/hhblits> with database: uniprot20_2013_03). The representative alignment was stored and used as a HMM model for HMM-HMM alignment performed with hhalign [77].

*GPCRDB*

Isberg et al. [24] performed a detailed structural comparison of the GPCR crystal structures and concluded that the optimal alignments of TMs between some pairs of GPCR proteins have single residue gaps, which correspond to bulges or constrictions on the helices. We downloaded the Isberg alignment from GPCRDB [78], which contains several differences from the published version in [24] (panels A, I, and N in Figure 3 of ref. [24]). We assume that the differences are caused by a need to reconcile the pairwise alignments into a global alignment.

To the best of our knowledge, the gaps in the GPCRDB alignment cannot be predicted without prior knowledge of the protein structures. For example, sequence CIGWG in CRF1 aligns to IG-WG in GLR (Fig. 3G in [24]). Also HMM-HMM has difficulty identifying the GPCRDB’s gaps. Table A highlights the differences between HMM-HMM and GPCRDB alignments in terms of number of misaligned residues in all TM regions for the crystallized GPCRs considered. HMM-HMM manages to predict all GPCRDB TM gaps for only a small number of target—template pairs (value 0 in Table A). However, for most pairs even within class A HMM-HMM cannot correctly determine the gaps (small positive values in the table). Large number of misaligned residues between different classes means that HMM-HMM is not suitable for comparisons across the different GPCR classes.

Table A. Number of misaligned residues in the TM regions between GPCRDB and HMM-HMM.

*Comparing GRoSS to the reference alignments*

The GRoSS alignment refines the BW TM.50 residues extended from class A to classes B, C, and F. In terms of the notation used in [24], this would be referred to as TM.50a, denoting the use of class A as a reference. For each TM and each target—template pair we compute the relative offset of the BW residues in the HMM-HMM alignment. If the offset is 0, we use “_” as a label; if the offset is 9 or more residues, we use “9”; and if template BW residue maps to a loop or a wrong TM, we use “X”. Table B shows the relative alignment of the BW residues between the GRoSS and the HMM-HMM alignments with the labels for the 7 TMs concatenated into one string.

When both the target and the template are from the same class, HMM-HMM aligns correctly all 7 BW .50 residues. When using class A templates for class B targets, HMM-HMM often gives the correct BW correspondence, and in some instances is off by one turn (4 residues) on TM5. Class C gets TM7 off by 6 residues, which already constitutes too large of an error for homology models. The alignment between class A and SMO (class F) varies, but most often it disagrees by 4 residues on TM5 and 1 residue on TM7.

Table C shows the same comparison but between the GRoSS and the GPCRDB alignments. Here, classes A and B are aligned identically, class C differs by 1 residue on TM7 and class F differs on 3 TMs by 2 or 3 residues.

Table B. Relative alignment of BW residues between GRoSS and HMM-HMM.

Table C. Relative alignment of BW residues between GRoSS and GPCRDB.

GRoSS, HMM-HMM and GPCRDB agree on alignment of the BW .50 residues for all TMs within class A. In this case, the only differences between these alignments are the gaps in HMM-HMM. Some gaps can be present in both target and template at matching locations, which would simplify homology modeling. Table D shows the number of residues aligned to gaps for each target—template pair when HMM-HMM is used. Similarly to the notation in the previous tables, we label “_” when there are no gaps; “9” for 9 or more gaps; “Y” for misaligned BW residues; “X” for any template residues aligned to loop regions of the target.

Table E shows the number of residues aligned to gaps if the GPCRDB alignment is used. Most target—template pairs have at least one gap. However, the number of gaps predicted by HMM-HMM (Table D) is larger and often falls at wrong positions, which disagree with GPCRDB (Table A).

Table D. Gaps in HMM-HMM: Number of residues in template TMs aligned to gaps in target sequence.

Table E. Gaps in GPCRDB: Number of residues in template TMs aligned to gaps in target sequence.

**Geometrical quality of homology models**

Each alignment can be used to produce a homology model for a given target—template pair. For the following analysis, we constructed simple homology models for the backbone atoms only. We ignored any missing residues, which were gaps in the target—template alignment. For all pairs considered in the previous tables, we evaluated RMSD, TM-score, and the number of common inter-helical contacts. The results of these three measures comparing HMM-HMM with GRoSS are shown in Figure A panels 1, 2, and 3. The same measures comparing GPCRDB with GRoSS are shown in Figure A panels 4, 5, and 6.

The RMSD comparisons (lower number is better) show that GRoSS alignment outperforms HMM-HMM for essentially all cases (Fig. A1). The RMSD comparisons of GRoSS to GPCRDB (Fig. A4), show that GRoSS outperforms GPCRDB in cross-class cases and only slightly underperforms in intra-class cases. This is expected as GPCRDB alignments include gaps/bulges based on pairwise structural comparison input, whereas GRoSS alignment ignores these gaps and bulges. TM-Score comparisons (higher number is better) show similar results as RMSD comparisons for GRoSS versus HMM-HMM (Fig. A2) and GRoSS versus GPCRDB (Fig. A5).

The comparison of the number of conserved contacts (higher number is better) shows that HMM-HMM performs slightly better than GRoSS (Fig. A3) for intra-class cases, but fails for inter-class cases. Same is true for GPCRDB comparison with GRoSS (Fig. A6).

Overall, these comparisons show that GRoSS alignments perform better than both HMM-HMM and GPCRDB. The cross-class sequence alignments for GRoSS are significantly better, whereas the intra-class sequence alignments are of similar quality.

Figure A. Comparing geometrical quality of the homology models.

| 1 | 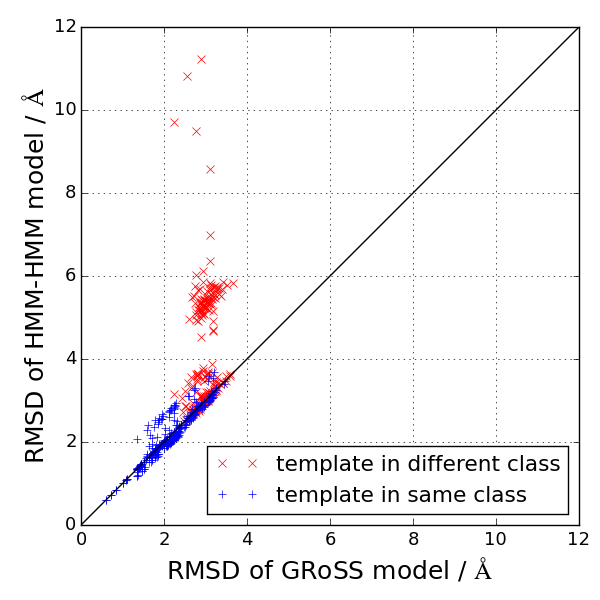 | 4 | 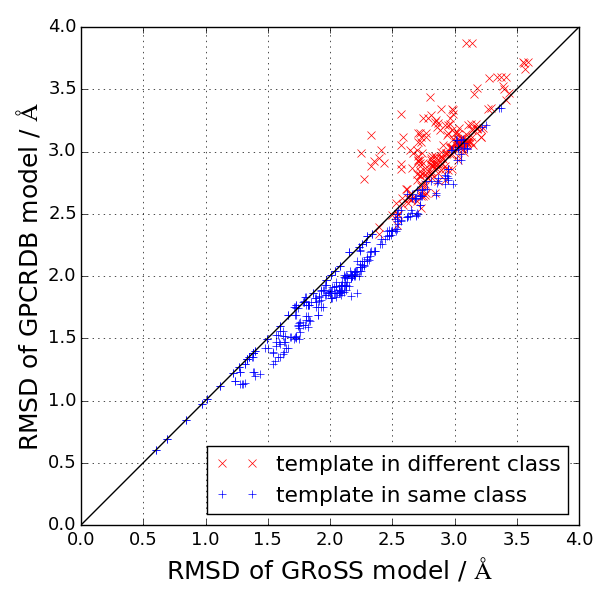 |
| --- | --- | --- | --- |
| 2 | 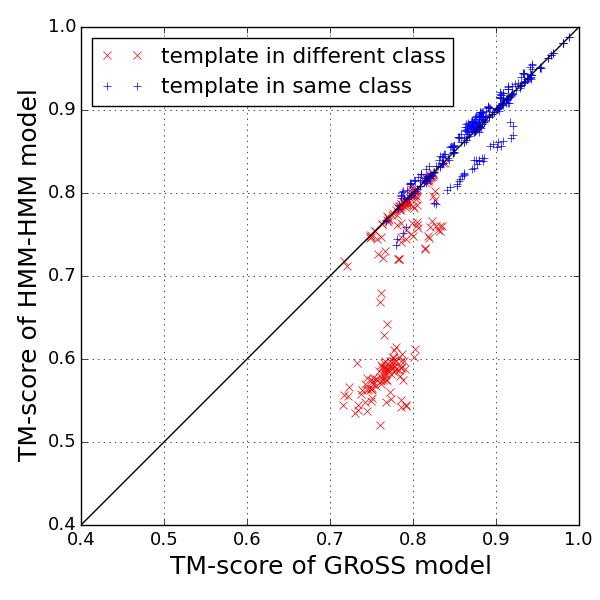 | 5 | 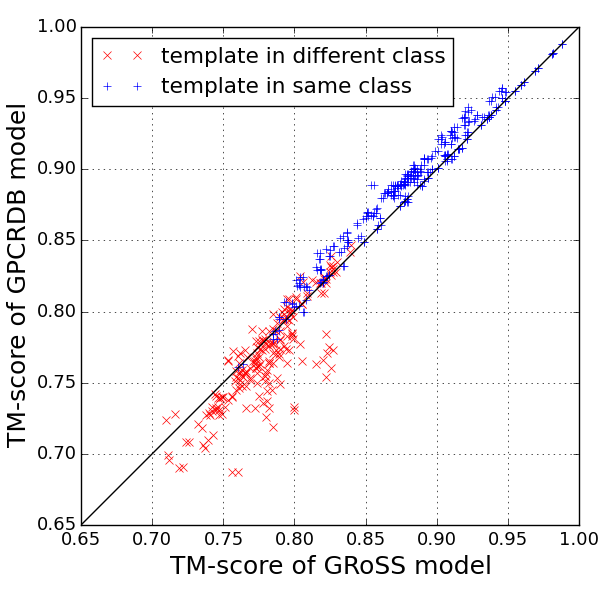 |
| 3 | 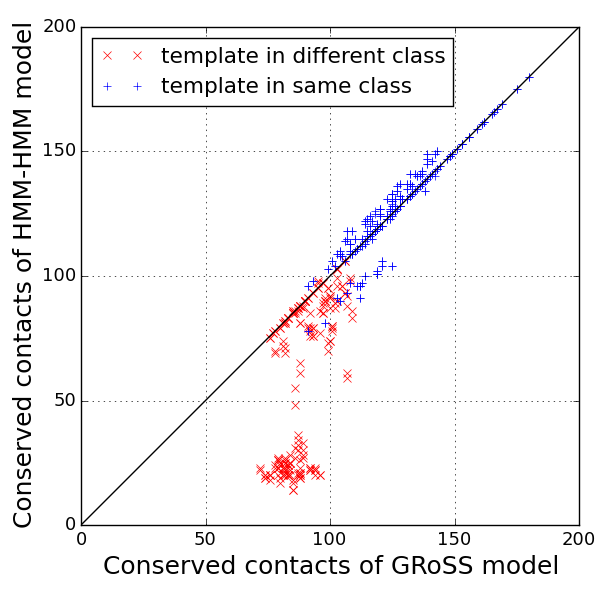 | 6 | 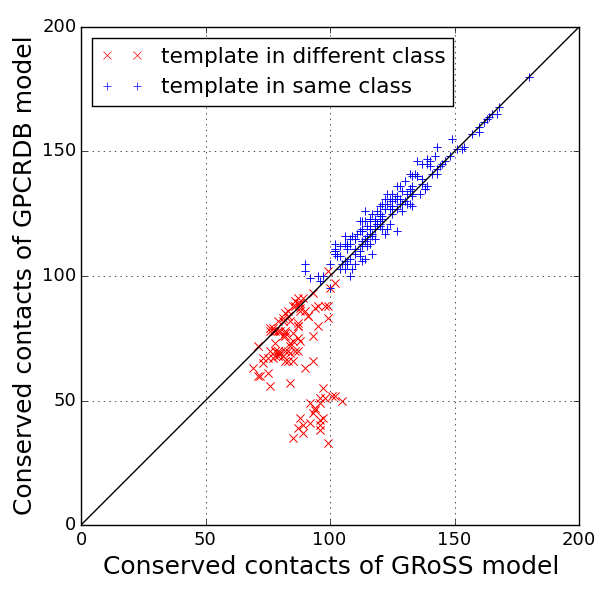 |
